# Expression Patterns of Genes Involved in Ascorbate-Glutathione Cycle in Aphid-Infested Maize (*Zea mays* L.) Seedlings

**DOI:** 10.3390/ijms17030268

**Published:** 2016-02-23

**Authors:** Hubert Sytykiewicz

**Affiliations:** Department of Biochemistry and Molecular Biology, Siedlce University of Natural Sciences and Humanities, Prusa 12, 08-110 Siedlce, Poland; huberts@uph.edu.pl; Tel.: +48-25-643-1298; Fax: +48-25-644-5959

**Keywords:** ascorbate, glutathione, antioxidative system, ascorbate-glutathione cycle, gene expression, *Rhopalosiphum padi*, *Sitobion avenae*, maize

## Abstract

Reduced forms of ascorbate (AsA) and glutathione (GSH) are among the most important non-enzymatic foliar antioxidants in maize (*Zea mays* L.). The survey was aimed to evaluate impact of bird cherry-oat aphid (*Rhopalosiphum padi* L.) or grain aphid (*Sitobion avenae* F.) herbivory on expression of genes related to ascorbate-glutathione (AsA-GSH) cycle in seedlings of six maize varieties (Ambrozja, Nana, Tasty Sweet, Touran, Waza, Złota Karłowa), differing in resistance to the cereal aphids. Relative expression of sixteen maize genes encoding isoenzymes of ascorbate peroxidase (*APX1*, *APX2*, *APX3*, *APX4*, *APX5*, *APX6*, *APX7*), monodehydroascorbate reductase (*MDHAR1*, *MDHAR2*, *MDHAR3*, *MDHAR4*), dehydroascorbate reductase (*DHAR1*, *DHAR2*, *DHAR3*) and glutathione reductase (*GR1*, *GR2*) was quantified. Furthermore, effect of hemipterans’ attack on activity of APX, MDHAR, DHAR and GR enzymes, and the content of reduced and oxidized ascorbate and glutathione in maize plants were assessed. Seedling leaves of more resistant *Z. mays* varieties responded higher elevations in abundance of target transcripts. In addition, earlier and stronger aphid-triggered changes in activity of APX, MDHAR, DHAR and GR enzymes, and greater modulations in amount of the analyzed antioxidative metabolites were detected in foliar tissues of highly resistant Ambrozja genotype in relation to susceptible Tasty Sweet plants.

## 1. Introduction

Ascorbic acid (AsA) and reduced glutathione (GSH; γ-l-glutamyl-l-cysteinylglycine) are major non-enzymatic and hydrophilic antioxidants in the leaves of maize (*Zea mays* L.) [1,2]. In the plant kingdom, the fundamental role of these compounds is linked to scavenging a broad range of reactive oxygen species (ROS) generated under physiological as well as oxidative stress conditions [3,4]. It has been signally reported that elevated amount of GSH in the stressed plants may influence the gene expression through thiol-mediated alternations in the structure of mitogen-activated protein kinases (MAPKs), transcription factors and RNA polymerase [5,6]. GSH is biosynthesized in plastids and cytosol, and it is being transported via unrecognized routes to the other subcellular locations [7], whereas AsA is produced in mitochondria, and subsequently, it is translocated to the other compartments or even extracellularly through the plasma membrane [8]. The content of these metabolites varies considerably between diverse plant systems, in dependence on genotype, developmental stage and organ, tissue and subcellular localization [3,7]. It has been evidenced that multifarious exogenous stimuli (e.g., heavy metals, ozone, salinity, pathogens or parasites) affect foliar amounts of GSH and/or AsA, and their redox ratios in the stressed plants [8,9]. There is a paucity of knowledge regarding the role of these antioxidants in complex interactions formulating between aphids and their hosts [10,11,12]. Furthermore, the intervarietal differences in the content of reduced and oxidized forms of glutathione and ascorbate (GSH and GSSG, AsA and DHA, respectively), as well as the ratios of GSH/GSSG and AsA/DHA in the aphid-infested plants were not reported so far.

Ascorbate-glutathione (AsA-GSH) cycle, also named as Foyer-Halliwell-Asada pathway, is one of the most crucial components of the antioxidative system in foliar tissues of the higher plants [3]. It participates in scavenging of hydrogen peroxide (H_2_O_2_), by utilizing AsA and GSH, thus affecting counteraction of the oxidative burst generated in response to adverse exogenous factors [13]. It has been identified four enzymes operating within the AsA-GSH cycle: ascorbate peroxidase (APX; EC 1.11.1.11), monodehydroascorbate reductase (MDHAR; EC 1.6.5.4), dehydroascorbate reductase (DHAR; EC 1.8.5.1), and glutathione reductase (GR; EC 1.8.1.7) [4,14]. In this pathway, the reduction of hydrogen peroxide is catalysed by APX, leading to production of monodehydroascorbate radical (MDHA) and water. The radical is converted to ascorbate in reaction catalysed by MDHAR. Alternatively, this ROS form may be disproportionated into dehydroascorbate (DHA), and subsequently, it can be reduced by DHAR to the ascorbic acid. GR catalyses reduction of oxidized glutathione (GSSG; glutathione disulphide) to two molecules of GSH [15]. At the biochemical level, it has been documented that a wide array of abiotic or biotic stressors (e.g., chilling, drought, heavy metals, salinity, exogenous application of abscisic acid (ABA) and H_2_O_2_, pathogen or parasite attack) may lead to substantial alternations in activity of the enzymes associated with the AsA-GSH cycle in several plant species [3,8]. However, transcriptional regulation of the genes related to the AsA-GSH pathway remains largely unknown [16,17]. Until now, there is no available published data regarding impact of the aphids’ feeding on expression of the genes encoding isozymes involved in course of the AsA-GSH cycle in tissues of either mono- or dicotyledonous plants. 

Aphids (Hemiptera: Aphidoidea) represent a diverse group of phloem-feeders, causing a broad range of detrimental effects within the colonized hosts [18,19]. An enhanced generation of various ROS forms in tissues of certain plant systems infested with a few aphid species has been signally reported [20,21,22]. Based on the results of previous experiments [21,22,23], it has been assumed that maize varieties, contrasting in resistance degrees to the cereal aphids’ attack, may respond differentially in expression levels of the genes involved in the AsA-GSH cycle and quantities of the analyzed antioxidants. Hence, the major objective of the performed molecular survey was aimed to assess the influence of two cereal aphid species (*i.e.*, oligophagous bird cherry-oat aphid, *Rhopalosiphum padi* L. or monophagous grain aphid, *Sitobion avenae* F.) infestation on relative expression of the sixteen genes encoding the isoenzymes related to the AsA-GSH cycle in the seedling leaves of maize. The first stage of the biotests was conducted on two *Z. mays* varieties: Ambrozja and Tasty Sweet (highly resistant and susceptible to the cereal aphids, respectively). The genetic studies encompassed measurements of the relative expression of *Z. mays* genes encoding isoenzymes of ascorbate peroxidase (*APX1*, *APX2*, *APX3*, *APX4*, *APX5*, *APX6* and *APX7*), monodehydroascorbate reductase (*MDHAR1*, *MDHAR2*, *MDHAR3* and *MDHAR4*), dehydroascorbate reductase (*DHAR1*, *DHAR2* and *DHAR3*), and glutathione reductase (*GR1* and *GR2*). In parallel, the total activity of APX, MDHAR, DHAR and GR enzymes were determined in the seedling leaves of Ambrozja and Tasty Sweet genotypes. In the second phase of the experiments, transcript abundance of four highly-regulated genes (*i.e.*, *APX1*, *MDHAR1*, *DHAR2* and *GR1*) was measured in the seedlings of six maize varieties (Ambrozja, Nana, Tasty Sweet, Touran, Waza and Złota Karłowa), massively infested with the investigated insects. The additional purpose of the current work was to evaluate the scale of intervarietal alternations in the amount of reduced and oxidized forms of glutathione and ascorbate, and the ratios of GSH/GSSG and AsA/DHA in foliar tissues of the aphid-injured maize plants. Furthermore, time-course and insect density-dependent changes in terms of both the abundance of the quantified transcripts and amount of the tested metabolites in the insect-stressed *Z. mays* seedlings were investigated.

## 2. Results and Discussion

### 2.1. Alternations in Glutathione Amount in Z. mays Seedlings Exposed to the Cereal Aphids’ Attack

**Figure 1:**
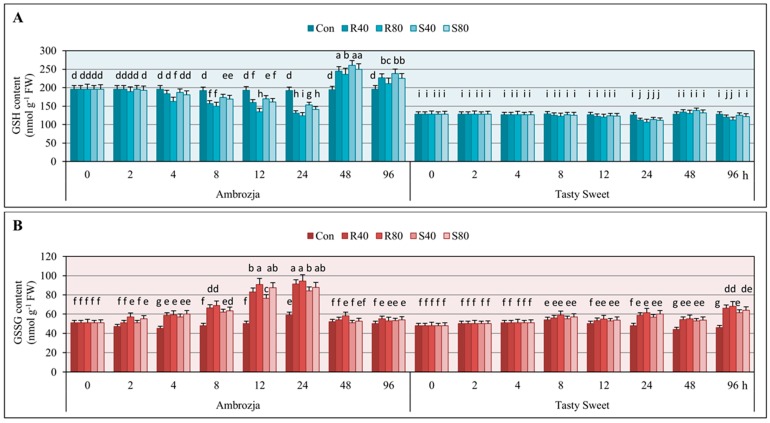
Effect of the cereal aphids’ infestation on the content of reduced glutathione (GSH) (**A**); and oxidized glutathione (GSSG) (**B**) in the seedlings of Ambrozja and Tasty Sweet maize cultivars. R40, R80—maize plants of a certain genotype infested with 40 or 80 *R. padi* females per seedling, accordingly; S40, S80—maize plants of a certain genotype infested with 40 or 80 *S. avenae* females per seedling, respectively. All data are presented as the mean (±SD) of three independent biotests, and significant differences between tested groups of the maize plants are denoted by different letters (*p* < 0.05; *post-hoc* Tukey’s test).

The performed experiments revealed that the seedlings of two investigated maize genotypes (Ambrozja and Tasty Sweet) infested with *R. padi* or *S. avenae* apterae (40 or 80 adult parthenogenetic females per plant), characterized with significant fluctuations in content of GSH in comparison with the uninfested controls (Figure 1). Furthermore, the largest changes in GSH levels occurred in the seedlings of more resistant Ambrozja cultivar, whereas the lowest alternations were noted in tissues of Tasty Sweet (susceptible) variety. Generally, concentration of the analyzed compound in *Z. mays* seedlings remained unaffected after 2-h feeding of the tested hemipterans. However, few exceptions were observed (*i.e.*, 1%–4% declines in GSH content in Ambrozja seedlings infested with 80 females of *R. padi* or *S. avenae* per plant). Doubling of the insect exposure time (up to 4 hpi) resulted in 5%–17% diminishes in GSH amount in Ambrozja plants, whereas Tasty Sweet seedlings did not respond any disturbances in GSH concentration compared to the uninfested control. After the next three time points (8, 12 and 24 hpi) of the cereal aphids’ infestation, a gradual depletion in content of the examined antioxidant in tissues of two tested maize genotypes was demonstrated. The greatest decreases in GSH level were noted in Ambrozja seedlings (10%–22% declines at 8 hpi; 12%–30% at 12 hpi; 25%–38% at 24 hpi), whereas the lowest depletion was recorded in Tasty Sweet plants (2%–5% declines at 8 hpi; 3%–8% at 12 hpi; 10%–15% at 24 hpi). Interestingly, 48-h insect feeding caused differential GSH accumulation in the maize seedlings (20%–34% and 2%–8% increases in Ambrozja and Tasty Sweet, accordingly). At 96 hpi, 2%–12% declines in GSH amount in Tasty Sweet plants were observed, while the reverse tendency in the seedlings of Ambrozja variety (*i.e.*, 9%–22% elevations) was demonstrated. Additionally, the higher impact of *R. padi* females’ attack on GSH concentration in the maize plants in comparison with the grain aphids was revealed. For example, infestation of *Z. mays* seedlings with 80 *R. padi* apterae per plant led to 5%–14% and 2%–7% greater declines in tissues of Ambrozja and Tasty Sweet cultivars, respectively, compared to the same number of *S. avenae* females. However, 48- and 96-h colonization of Ambrozja seedlings, as well as 48-h infestation of Tasty Sweet seedlings with *S. avenae* females, evoked slightly higher increments in amount of the analyzed metabolite in relation to GSH levels in the respective plants attacked by the bird cherry-oat aphid females. Interestingly, lower number of aphids (40 females of *R. padi* or *S. avenae* per plant) led to higher increases in GSH content in the leaves of two tested maize varieties in relation to the higher infestation level (80 insects per seedling). Additionally, negligible differences in GSH amount in the non-stressed (control) seedlings of the investigated *Z. mays* cultivars were recorded throughout the experimental periods (0‒96 hpi).

Results of the biotests assessing the effect of *R. padi* or *S. avenae* infestation on GSSG amount in the seedlings of Ambrozja and Tasty Sweet genotypes are depicted in Figure 1. It was revealed a gradual increase in the level of oxidized glutathione from 2 hpi (5%–12%) to 24 hpi (65%–85%) in Ambrozja plants, and from 8 hpi (2%–10%) to 24 hpi (18%–28%) in tissues of Tasty Sweet cultivar. At 48 hpi, lower increments in GSSG level were recorded (1%–8% and 10%–15% in Ambrozja and Tasty Sweet seedlings, respectively). Importantly, prolonged aphid feeding (96 hpi) was linked to strong elevation (26%–42%) in GSSG content in Tasty Sweet plants, and only minor increments in the quantified compound (4%–11%) were detected in seedlings of Ambrozja genotype. Moreover, *R. padi* females caused more circumstantial increases in GSSG amount (3%–26% and 2%–12% higher elevations in Ambrozja and Tasty Sweet plants, accordingly) in relation to the grain aphids. The opposite effect was noted in Ambrozja seedlings colonized by 80 *S. avenae* females per plant (at 4 hpi).

It has been also demonstrated that the cereal aphid’s infestation led to substantial decreases in GSH/GSSG ratios in tissues of two tested maize cultivars (Supplementary Material, Figure S1). It was observed an earlier and higher decrement in the GSH/GSSG ratio in tissues of highly resistant Ambrozja cultivar compared to the susceptible one. The level of the estimated parameter declined progressively from 2 to 24 hpi (8%–15% to 47%–61%, respectively) in Ambrozja plants, and decreased from 8 to 24 hpi (4%–13% to 26%–34%, accordingly) in Tasty Sweet seedlings. Long-term cereal aphids’ infestation was associated with elevations in GSH/GSSG ratios in Ambrozja plants (13%–37% at 48 hpi; 3%–15% at 96 hpi), but converse responses were revealed in seedling leaves of Tasty Sweet variety (10%–19% decreases at 48 hpi; 26%–41% declines at 96 hpi). Furthermore, it was evidenced a higher impact of the bird cherry-oat aphids’ feeding on level of the GSH/GSSG ratio (*i.e.*, 3%–16% and 2%–9% higher alternations in Ambrozja and Tasty Sweet plants, respectively) in comparison with *S. avenae* apterae.

Analysis of variance (factorial ANOVA) proved significance of the tested variables (maize genotype, aphid species, aphid abundance and infestation time) and their interactions on the content of GSH and GSSG, as well as the ratio of GSH/GSSG in *Z. mays* seedlings (Supplementary Material, Tables S1 and S2). 

Glutathione is a well described antioxidant containing a cysteinyl thiol group, involved in maintaining the intracellular redox state in plants [5]. In addition, it has been reported a crucial role of GSH in detoxification of xenobiotics, heavy metals’ tolerance, regulation of ROS signaling and antioxidative responses to multifarious environmental stresses in planta [24,25,26]. In the current work, it has been uncovered that the aphid-infested leaves of more resistant maize genotype (cv. Ambrozja) reacted a greater progressive depletion in GSH content and the redox ratio of GSH/GSSG as well as elevation in GSSG amount (up to 24 hpi), and conversely, significantly higher accumulation of GSH and level of GSH/GSSG ratio at 48 and 96 hpi in relation to the susceptible one (cv. Tasty Sweet). It indicated the pivotal role of GSH in conferring enhanced resistance of *Z. mays* cultivars against the cereal aphids’ infestation. Schlaeppi *et al.* [27] documented that GSH-deficient *pad2-1* mutant exhibited a higher susceptibility to the generalist insect Egyptian cotton worm (*Spodoptera littoralis* Boisduval) (Lepidoptera: Noctuidae) in relation to the wild-type (WT) plants. According to these authors, deficiency in the GSH pool in *Arabidopsis thaliana* L. resulted in suppressed resistance toward the insects in the infested plants, and a substantial deceleration in biosynthesis of glucosinolates (*i.e.*, 4-methylsulfinylbutyl-glucosinolate and indolyl-3-methyl-glucosinolate) in response to *S. littoralis* infestation. In addition, Liu *et al.* [14] demonstrated that total glutathione content progressively increased at the feeding sites of the Hessian fly (*Mayetiola destructor* Say) (Diptera: Cecidomyiidae) in the wheat seedlings of resistant (Molly) genotype in comparison with the non-stressed plants. The insect-evoked enhancement in amount of the total glutathione occurred in these wheat plants from 3 to 72 hpi, reaching a 60% increase at the last examined period of the experiments. Importantly, it has been recorded only slight fluctuations in concentration of the analyzed antioxidant in tissues of susceptible wheat (cv. Newton) plants infested with *M. destructor*. In contrast, Kar *et al.* [9] revealed that leaves of *Terminalia arjuna* Arjun infested with sap-sucking *Trioza fletcheri* Crawford (Hemiptera: Psyllidae) responded a significant decline in GSH concentration in comparison with the untreated control. Recently, Matern *et al.* [6] ascertained that the transgenic lines of tobacco (*Nicotiana tabacum* L.) containing high level of GSH characterized with an enhanced resistance to *Pseudomonas syringae* (pathovars *maculicola* and *tabaci* 11528). Several defence reactions were recognized, e.g., increased callose formation and deposition, higher accumulation of salicylic acid (SA), more severe hypersensitive (HR) reactions and upregulation of numerous genes encoding the pathogenesis-related proteins (*i.e.*, *PR1*, *PR2*, *PR4* and *PR5*) [6]. It has also been evidenced that MAPKs and SA signaling pathways may be affected by the glutathione redox state. Additionally, Matern *et al.* claimed that concentration of reduced or oxidized forms of glutathione profoundly affected many physiological processes, activity of enzymes and expression of several genes in planta [6]. However, detailed mechanisms underlying direct and/or indirect genome-wide reconfigurations in expression of glutathione-dependent genes in plant systems still remain to be deciphered.

According to Dubreuil-Maurizi and Poinssot [28], phytoalexin-deficient *pad2-1* mutant of *A. thaliana* is characterized with a low GSH content (approx. 20% of the amount in the WT plants) that secondarily disturbs functioning of the complex signaling networks in response to phytophage attack. It has been elucidated by these authors, that *pad2-1* mutant displayed an elevated susceptibility to infestation with *S. littoralis* as well as colonization by as a wide array of fungal or bacterial pathogens. Additionally, Koffler *et al.* [29] revealed that *pad2-1* mutant of *A. thaliana* was more susceptible to cadmium (Cd) treatment in comparison with Col-0 plants. Detailed analyses proved that non-stressed *pad2-1* plants possessed 71% and 87% lower contents of reduced glutathione in chloroplasts and nuclei, accordingly, compared to the WT controls. The *pad2-1* mutant plants responded stronger decreases in GSH concentration as well as earlier occurrence of chlorosis and necrosis in Cd-treated plants than in the Col-0 ones. Therefore, it has been postulated that GSH contributes to higher protection of plants against excessive accumulation of ROS and oxidative stress-related damages. Microarray experiments performed by Kumar *et al.* [5] evidenced significant upregulation of 973 and downregulation of 701 transcripts in *A. thaliana pad2-1* plants exposed to combined osmotic and cold stresses. Comparative transcriptomic investigations revealed downregulation of genes associated with biogenesis of ethylene, lignin and phenylpropanoids in the stressed plants. Furthermore, expression of a wide array of genes encoding various transcriptional factors (e.g., ARR-B, C2C2-CO-like, HSF, MADS, MYB-related and NAC) were found to be markedly repressed in *pad2-1* plants in relation to the WT ones. In addition, proteomic approach identified that approx. 64% of the downregulated proteins (e.g., lycopene β-cyclase, glutathione transferase, heat shock protein-70, peptidyl-prolyl isomerase and NBS-LRR type resistance protein) in *pad2-1* mutant were linked to diverse defence reactions, indicating the involvement of GSH molecules in the sophisticated cross-talk between SA and ethylene signaling routes in *A. thaliana* plants.

### 2.2. Aphid-Induced Changes in Ascorbate Content in the Maize Plants

**Figure 2:**
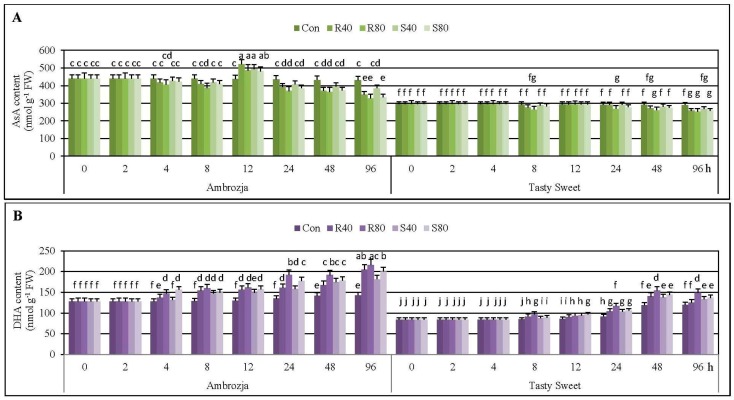
Influence of the cereal aphid’s infestation on the content of reduced ascorbate‒AsA (**A**); and oxidized ascorbate‒DHA (**B**) in the seedlings of Ambrozja and Tasty Sweet maize cultivars. R40, R80—maize plants of a certain genotype infested with 40 or 80 *R. padi* females per seedling, accordingly; S40, S80—maize plants of a certain genotype infested with 40 or 80 *S. avenae* females per seedling, respectively. All data are presented as the mean (±SD) of three independent biotests, and significant differences between tested groups of the maize plants are denoted by different letters (*p* < 0.05; *post-hoc* Tukey’s test).

Results regarding the effect of the cereal aphids’ herbivory on concentration of the reduced and oxidized ascorbate (AsA and DHA, respectively) in the seedling leaves of two tested *Z. mays* genotypes (Ambrozja and Tasty Sweet) are presented in Figure 2.

It has been elucidated that 2-h feeding of *R. padi* or *S. avenae* females did not cause any disturbances in content of AsA in seedlings of the investigated maize genotypes, compared to the uninfested controls. Likewise, 4-h colonization of Tasty Sweet plants with the tested hemipterans did not evoke any alternations in AsA concentration. However, 3%–8% decreases in amount of the quantified antioxidant in tissues of the aphid-stressed Ambrozja plants were recorded. Doubling of the insect exposure time (up to 8 hpi) resulted in quite similar declines in AsA levels in the maize seedlings (5%–12% and 2%–10% decreases in Ambrozja and Tasty Sweet varieties, accordingly). It is noteworthy, that extending of the aphid infestation time up to 12 hpi was linked to 10%–19% elevations in AsA content in Ambrozja seedlings. Additionally, at this time point, amount of the analyzed compound in Tasty Sweet plants remained unaffected, compared to the control. At 24 hpi, the cereal aphids’ infestation led to depletion in AsA concentration in the leaves of more resistant Ambrozja variety (8%–15% decreases; depending on the specific aphid treatment). Furthermore, lower abundance of the insects (40 females of *R. padi* or *S. avenae* per seedling) did not alternate AsA amount in Tasty Sweet plants, while higher number of the hemipterans (80 insects per seedling) caused 5%–8% declines in concentration of the examined compound. The next studied period of the cereal aphids’ colonization (48 hpi) was associated with depletion in AsA foliar pool in the seedlings of two investigated maize genotypes (11%–18% and 4%–11% decreases in Ambrozja and Tasty Sweet cultivars, respectively). After 96-h feeding of the tested insects, greater diminution in amount of the antioxidant occurred in tissues of Ambrozja plants (10%–24% declines) in relation to the aphid-susceptible Tasty Sweet genotype (6%–13% decreases). Generally, it has also been demonstrated that *R. padi* apterae provoked slightly greater changes in AsA concentration in the seedlings of the tested maize cultivars in comparison with the grain aphids. Furthermore, a tendency toward a slight reduction in AsA amount in the uninfested seedlings of two examined maize varieties during the tested periods of the biotests (0–96 hpi) has been uncovered.

Furthermore, it has been established that the cereal aphids’ infestation stimulated accumulation of DHA in the seedling leaves of two examined maize cultivars (Figure 2). It was demonstrated a steady increase in DHA level in Ambrozja plants, from 4 to 96 hpi (5%–20% to 28%–55%, respectively). Aphid-stressed Tasty Sweet seedlings responded lower and gradual increments in DHA content, from 8 to 48 hpi (3%–14% to 16%–30%, accordingly), but further extending of the hemipterans’ colonization (up to 96 hpi) caused lesser increments (9%–24%) in amount of the analyzed constituent. It is important to underline, that *R. padi* apterae affected 2%–16% and 1%–10% higher increases in DHA amount in seedlings of Ambrozja and Tasty Sweet varieties, respectively. On the contrary, Ambrozja plants challenged by 40 or 80 *S. avenae* females per seedling (4 and 48 hpi), and Tasty Sweet plants attacked by 40 or 80 apterae of the same insect species (12 and 96 hpi), possessed higher contents of DHA in relation to the maize plants infested with the bird cherry-oat aphids. In addition, it has been detected minor increases in level of DHA in the non-stressed maize plants throughout the experimental periods.

Time-course analysis evidenced circumstantial declines in AsA/DHA ratios in aphid-stressed Ambrozja and Tasty Sweet plants (2%–50%, and 4%–32% decrements, respectively) (Supplementary Material, Figure S1). The earlier decrease in AsA/DHA ratio was observed in Ambrozja plants (at 4 hpi, 6%–20% declines), while the level of examined parameter in seedlings of the other tested genotype started to decline at 8 hpi (5%–22%). The highest diminution in AsA/DHA ratio in the seedlings of both maize varieties were recorded at 48 hpi (25%–38% declines—Ambrozja; 16%–30%—Tasty Sweet), and after 96 hpi (29%–50% decreases—Ambrozja; 18%–32%—Tasty Sweet). It has also been evidenced a more substantial influence of the bird cherry-oat aphids’ herbivory on AsA/DHA ratios in the maize seedlings (6%–15% and 2%–13% higher changes in Ambrozja and Tasty Sweet, accordingly) in relation to *S. avenae* attack. However, few exceptions were identified: Ambrozja plants infested with 80 or 40 grain aphids per plant (4 and 12 hpi), and Tasty Sweet seedlings attacked by 40 or 80 apterae per seedling (12 and 96 hpi), characterized with slightly higher declines in AsA/DHA ratio, compared to the respective groups of maize plants colonized by *R. padi* females.

Statistical analysis proved the significant influence of the examined parameters and their interconnections on the amount of both tested forms of ascorbate (AsA and DHA), and the ratio of AsA/DHA in the maize seedlings (Supplementary Material, Tables S1 and S2).

Ascorbate is a multifunctional hydrophilic antioxidant involved in detoxification of diverse ROS forms, regeneration of tocopherols, redox signaling as well as complex regulation of several physiological processes (e.g., flowering and senescence) and transcriptional activity of a wide range of genes in higher plants. It also serves as a cofactor of many biocatalysts involved in photosynthesis process, biogenesis of phytohormones and flavonoids [30,31]. Furthermore, it has been reported that AsA participates in triggering of defence reactions against a broad spectrum of abiotic and biotic stressing factors [26,32]. In the present study, more resistant maize genotype (cv. Ambrozja) exhibited the elevated content of AsA at 12 hpi, while no response or slight diminution in amount of this metabolite was detected in tissues of the susceptible one. Additionally, prolonged infestation of the cereal aphids caused larger declines in AsA concentration and AsA/DHA redox ratio, and conversely, higher increments in DHA content in Ambrozja seedlings, compared to Tasty Sweet plants. Aphids do not produce AsA in their bodies, hence, sufficient level of dietary ascorbate has to be provided by the host tissues [12]. Local decline in AsA concentration at the aphids’ feeding site may restrain hemipterans’ performance [33]. Therefore, it is probable that the observed depletion in AsA content, and elevation in the oxidized pool of ascorbate in the leaves of more resistant maize variety in response to the cereal aphids’ colonization may be a component of specific defence strategy involving deterioration of nutritive value of the host. On the other hand, depletion in the AsA pool may be associated with utilizing of this compound as a substrate in the reaction catalysed by APX, thus regulating the content of H_2_O_2_ in the insect-challenged maize seedlings. Another possible explanation for the observed disturbances in AsA amount in the aphid-treated maize plants is downregulation of the genes involved in ascorbate biogenetic pathways. It should be underlined that particular mechanisms regulating AsA biosynthesis in tissues of higher plants remains largely unknown [34]. Kerchev *et al.* [12] revealed that AsA-deficient *vtc2-1* mutant of *A. thaliana* exhibited an activated SA-dependent signaling pathways, and simultaneously, displayed an enhanced resistance to the green peach aphid (*Myzus persicae* Sulzer), compared to the untransformed (Col-0) plants. In response to infestation with adult apterous of *M. persicae*, expression of 532 genes was upregulated, and 226 transcripts were downregulated in *vtc2-1* mutant in relation to the stressed WT plants. Although the insect fecundity was repressed when feeding on *vtc2-1* mutants, the number of aphids growing on the double mutant *abi4vtc2* (deficient in abscisic acid signaling, and also characterized with a reduced content of AsA) was slightly higher in respect to the Col-0 genotype. It provided evidence that AsA depletion may markedly regulate redox signaling-related defensive responses of the host plant toward aphids’ infestation, that secondarily leads to enhancement in the resistance level against these insects. Furthermore, these authors elucidated a crucial role of abscisic acid-insensitive 4 (ABI4) transcription factor and ascorbate-dependent receptor-like kinases in triggering of aphid-induced defence mechanisms in *A. thaliana* plants. Furthermore, Kerchev *et al.* [11] revealed that *M. persicae* attack (60 adult wingless females per leaf) after 8, 24 or 48 hpi did not affect significant alternations in AsA content in the leaves of potato (*Solanum tuberosum* L.) in comparison with the uninfested control. However, considerably higher concentration of DHA was detected at 48 hpi in the stressed leaves. In addition, approx. 2-fold higher abundance of aphids was observed on the plants treated with 50 mM l-galactono-1,4-lactone (essential precursor for ascorbate biogenetic route), compared to those treated with water. In the present study, it has been established a marginal decline in contents of AsA and DHA in the unstressed (control) seedlings of the examined maize varieties throughout the experimental periods (0–96 hpi). Similarly, Sanahuja-Solsona [35] ascertained that young leaves of maize (cv. B73) possessed a higher concentration of total and reduced ascorbate, and conversely, lower DHA amount in comparison with the mature ones. According to this author, greater AsA pool in the young leaves of maize was a result of upregulation of expression of the several genes related to l-galactose pathway, leading to an accelerated biosynthesis of the reduced ascorbate.

### 2.3. Insect-Affected Modulations in Expression of Ascorbate Peroxidase (APX) Genes and APX Activity in Z. mays Seedlings

**Figure 3:**
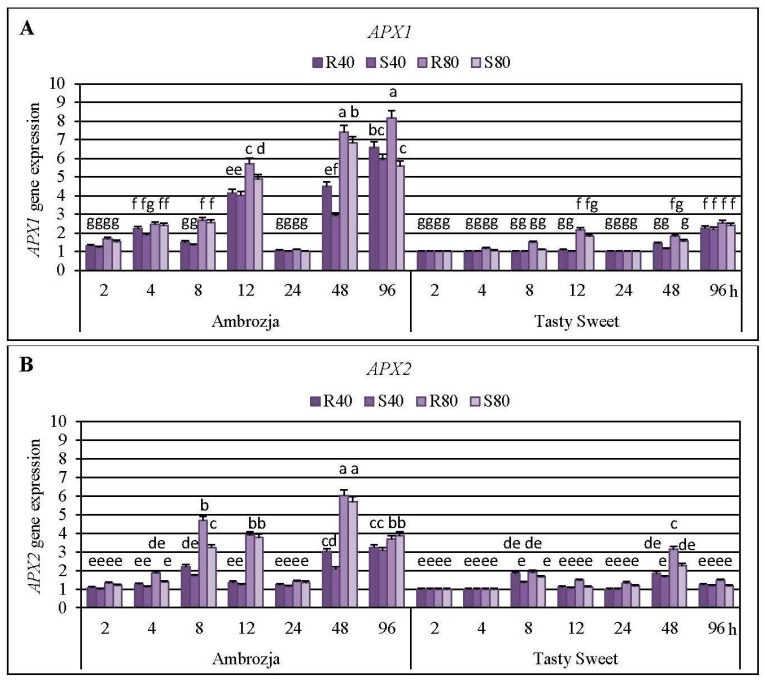
Effect of the cereal aphid’s herbivory on relative expression of *APX1* (**A**) and *APX2* (**B**) genes in the seedlings of Ambrozja and Tasty Sweet maize varieties (first phase of the experiments). R40, R80—maize plants of a certain genotype infested with 40 or 80 *R. padi* females per seedling, accordingly; S40, S80—maize plants of a certain genotype infested with 40 or 80 *S. avenae* females per seedling, respectively. All data are presented as the mean (±SD) of three independent biotests, and significant differences in abundance of the *APX* transcripts are denoted by different letters (*p* < 0.05; *post-hoc* Tukey’s test).

**Figure 4:**
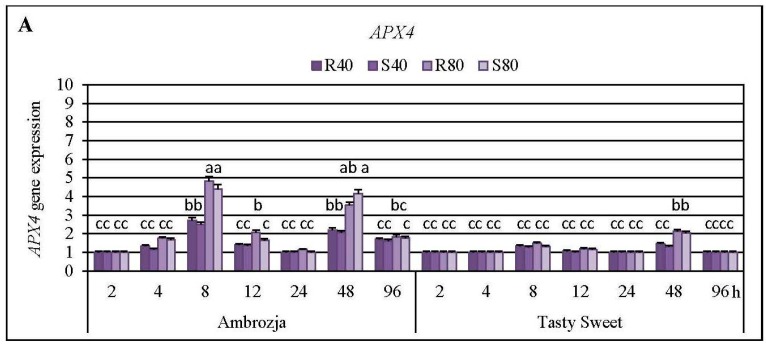

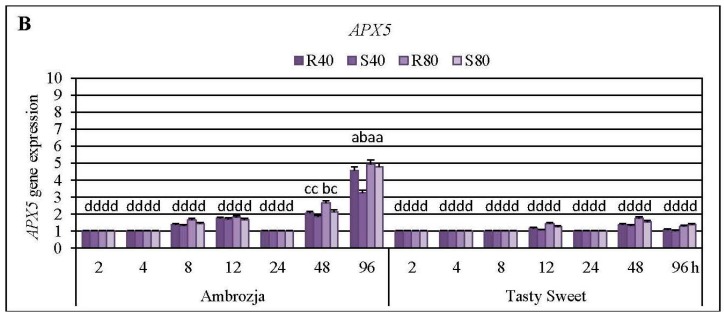
Influence of the cereal aphid’s infestation on relative expression of *APX4* (**A**) and *APX5* (**B**) genes in the seedlings of Ambrozja and Tasty Sweet maize varieties (first phase of the experiments). R40, R80—maize plants of a certain genotype infested with 40 or 80 *R. padi* females per seedling, accordingly; S40, S80—maize plants of a certain genotype infested with 40 or 80 *S. avenae* females per seedling, respectively. All data are presented as the mean (±SD) of three independent biotests, and significant differences in abundance of the *APX* transcripts are denoted by different letters (*p* < 0.05; *post-hoc* Tukey’s test).

**Figure 5:**
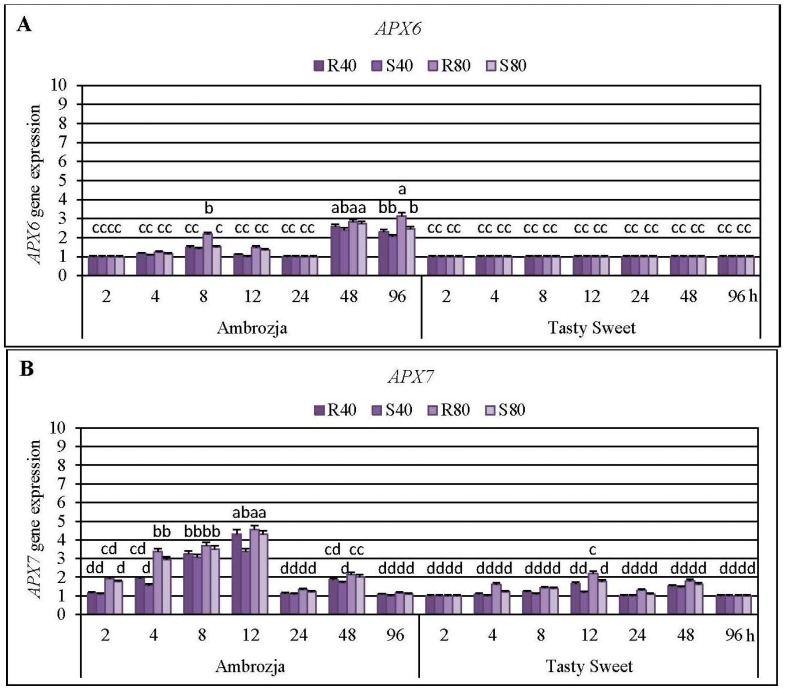
Impact of the cereal aphid’s attack on relative expression of *APX6* (**A**) and *APX7* (**B**) genes in the seedlings of Ambrozja and Tasty Sweet maize varieties (first phase of the experiments). R40, R80—maize plants of a certain genotype infested with 40 or 80 *R. padi* females per seedling, accordingly; S40, S80—maize plants of a certain genotype infested with 40 or 80 *S. avenae* females per seedling, respectively. All data are presented as the mean (±SD) of three independent biotests, and significant differences in abundance of the *APX* transcripts are denoted by different letters (*p* < 0.05; *post-hoc* Tukey’s test).

Cereal aphids’ colonization evoked differential patterns of the transcriptional responses of all seven *APX* genes in the seedlings of Ambrozja (highly resistant) and Tasty Sweet (susceptible) genotypes (Figure 3, Figure 4 and Figure 5; Supplementary Material, Figure S2). Among the quantified *Z. mays APX* transcripts, the strongest accumulation of *APX1* and *APX2* mRNA has been detected (5%–715% and 8%–504% elevations, respectively), abundance of *APX4*, *APX5*, *APX6* and *APX7* transcripts were moderately stimulated (7%–382%, 3%–395%, 4%–214% and 6%–355% increments, correspondingly), whereas the lowest increases (7%–76%) in expression of *APX3* gene were noted. Insect-infested Ambrozja plants characterized with a higher enhancement in expression of all the tested *APX* genes in relation to Tasty Sweet genotype. It should be emphasized that the cereal aphids’ infestation led to the significant upregulation of six *APX* genes (*APX1*, *APX2*, *APX4*, *APX5*, *APX6* and *APX7*) in Ambrozja (highly resistant) plants, whereas Tasty Sweet (susceptible) seedlings reacted the elevation in amount of four *APX* transcripts (*APX1*, *APX2*, *APX4* and *APX7*). In addition, Ambrozja seedlings responded an earlier increase in abundance of the examined transcripts than the other investigated maize cultivar. The highest upregulation of *APX1* gene in the aphid-infested Ambrozja seedlings occurred at 48 and 96 hpi (192%–641% and 460%–715% increases, respectively), while the maximal expression of the target gene in Tasty Sweet variety was noted at 96 hpi (120%–155% increases). The greatest abundance of *APX2* transcript in the maize seedlings exposed to the hemipterans’ attack was noted at 48 hpi (108%–504% and 65%–214% elevations in Ambrozja and Tasty Sweet plants, respectively). Similarly, the highest accumulation of *APX3* mRNA occurred at 48 hpi in Ambrozja plants (11%–76% increases), but expression of the target gene in Tasty Sweet seedlings remained unchanged during the experimental periods of the insect’s infestation (2–96 hpi). Furthermore, the maximal rise in *APX4* mRNA level was detected at 8 hpi in Ambrozja plants, and after 48 hpi in the leaves of Tasty Sweet genotype (150%–382% and 33%–112% increases, respectively). The highest increment (224%–395%) in *APX5* gene expression in Ambrozja seedlings was demonstrated at 96 hpi, whereas the greatest elevation (32%–93%) in the transcript amount in Tasty Sweet plants occurred at 48 hpi. Furthermore, the strongest upregulation of *APX6* gene was noted at 48 and 96 hpi in Ambrozja genotype (138%–182% and 105%–214% increments, respectively), but Tasty Sweet plants did not react any reconfigurations in abundance of *APX6* transcript from 2 to 96 hpi. It has also been revealed that the maximal increases (232%–355%) in *APX7* mRNA level in Ambrozja plants occurred at 12 hpi, whereas the highest elevations in Tasty Sweet variety were observed at 12 and 48 hpi (19%–120% and 45%–82% increases, correspondingly). In addition, it was found that *R. padi*-infested maize seedlings exhibited greater increments in expression of *APX1*, *APX2*, *APX3*, *APX4*, *APX5*, *APX6* and *APX7* genes (5%–259%, 7%–144%, 2%–26%, 6%–43%, 5%–131%, 4%–68%, and 2%–100% higher increases, respectively), compared to plants colonized by the grain aphids. However, few exceptions were identified (*i.e.*, Ambrozja plants treated with 80 *S. avenae* females per seedling possessed a 16% higher increment in *APX2* gene expression at 96 hpi, as well as this group of the seedlings displayed a 64% greater increase in *APX4* mRNA level at 48 hpi than those infested with the same number of *R. padi* females). Additionally, 96-h feeding of 80 *S. avenae* apterae per plant caused an 8% higher elevation in amount of *APX5* transcript in Tasty Sweet seedlings in comparison with *R. padi*-attacked plants. An insect density-dependent alternation in the quantity of the *APX* transcripts in *Z. mays* seedlings has also been revealed. A higher number of the cereal aphids (80 *R. padi* or *S. avenae* females per seedling) influenced more circumstantial increments in expression of all the studied *APX* genes (8%–391%, 5%–360%, 7%–59%, 4%–211%, 6%–154%, 8%–85% and 10%–143% greater increases in expression of *APX1*, *APX2*, *APX3*, *APX4*, *APX5*, *APX6* and *APX7* genes, accordingly), compared to the maize plants colonized by lower abundance of the tested hemipterans (40 insects per seedling). However, two exceptions were ascertained; Ambrozja plants attacked by 40 grain aphid females per plant responded a 33% higher increase in amount of *APX1* mRNA (at 96 hpi), and a 5% greater increment in *APX5* gene expression (at 12 hpi) in respective to the changes affected by 80 *S. avenae* aphids per seedling. 

The second stage of the biotests demonstrated a stimulation of *APX1* gene expression in tissues of all six investigated maize cultivars after 12 and 48 hpi (87%–482% and 9%–625% increases, accordingly, in dependence on the specific biotest variant) (Supplementary Material, Figure S3). The strongest upregulation of *APX1* gene at these two time points occurred in Ambrozja and Waza seedlings (410%–558% and 281%–625% elevations, accordingly). On the other hand, the lowest increment in *APX1* mRNA abundance was observed in Tasty Sweet and Złota Karłowa plants (9%–132% and 14%–180% increases, respectively). Additionally, low elevation in *APX1* gene expression was recorded at 48 hpi in Nana and Touran plants infested with *S. avenae* females (20% and 36% increases, respectively). At 24 hpi, amount of the analyzed transcript in almost all groups of the aphid-stressed maize plants was comparable to the respective uninfested plants; the only exception was the group of Waza seedlings treated with *R. padi* females that possessed an insignificantly greater level (*ca.* 22%) of *APX1* mRNA in relation to the non-stressed control. Furthermore, higher upregulation of the target gene occurred in the maize seedlings infested with the bird cherry-oat aphids in relation to *S. avenae*-attacked plants (45%–137% greater increases at 12 hpi, and 5%–232% increases at 48 hpi, depending on the tested maize genotype). Conversely, Tasty Sweet plants colonized by *S. avenae* females for 48 h, responded to a slightly higher elevation (*ca.* 6%) in expression of *APX1* gene, compared to the respective seedlings treated with *R. padi* aphids. 

A stimulation in total activity of APX enzyme has been found at five time intervals (4, 8, 12, 48 and 96 hpi) of the hemipterans’ infestation in Ambrozja seedlings, and after three time points (8, 48 and 96 hpi) in Tasty Sweet plants (Supplementary Material, Figure S4). Maximal enhancement of AXP activity in the leaves of both tested maize cultivars was recorded at 8 hpi (39%–62% and 21%–32% increases in Ambrozja and Tasty Sweet seedlings, respectively). Importantly, the aphid-stressed Ambrozja plants responded 11%–19% declines in total activity of APX enzyme at 24 hpi, whereas levels of the estimated parameter at this time point in Tasty Sweet seedlings were comparable to those in the controls. In addition, a more substantial impact of *R. padi* females on APX activity (*i.e.*, 3%–26% higher alternations) in tissues of both examined maize varieties in comparison with the grain aphids has been evidenced. However, two exceptions were noted; Ambrozja seedlings colonized by 40 and 80 *S. avenae* females per plant possessed about 7% higher increase at 96 hpi and 5% greater decrease at 24 hpi, respectively, in the enzyme activity, compared to *R. padi-*attacked plants.

Statistical analysis (factorial ANOVA) confirmed that the relative expression of six examined *APX* genes (*APX1*, *APX2*, *APX4*, *APX5 APX6* and *APX7*) in the maize seedlings was markedly influenced by all the tested variables and their interrelations (Supplementary Material, Tables S3–S5). However, transcriptional responses of *APX5* gene were not significantly affected by the interaction of three variables: maize genotype × aphid species × aphid abundance (*F*_2, 192_ = 1.6; *p* = 0.258) (Supplementary Material, Table S4). In addition, all the changes in expression of *APX3* gene in *Z. mays* seedlings were found to be insignificant (Supplementary Material, Table S3). Furthermore, all the examined indicators and their interactions significantly influenced the total activity of APX enzyme in the maize plants (Supplementary Material, Table S6).

AsA-GSH cycle is one of the most important antioxidative mechanisms involved in detoxification of excessive quantities of hydrogen peroxide generated in higher plants under optimal and oxidative stress-inducing environmental conditions [4,13]. APX is an integral element of the AsA-GSH cycle participating in decomposition of H_2_O_2_ to DHA and water, by utilizing the reduced ascorbate as an electron donor [36]. Until now, it has been identified seven *Z. mays APX* genes (*APX1*-*APX7*), encoding the respective isoforms (*i.e.*, cytosolic APX1, APX2 and APX4; peroxisomal APX3; mitochondrial APX5 and APX6; chloroplastic APX7) [16]. The presence of a wide spectrum of organelle-specific APX isoenzymes enables robust functioning of the AsA-GSH cycle in order to mitigate the exaggerated oxidative-relative damages in particular compartments in the plant cells [37]. Importantly, the structure of APX isoforms is quite unstable under AsA deficiency, therefore, the prompt regeneration and/or *de novo* biosynthesis of this low-molecular antioxidant are essential factors in maintaining the optimal H_2_O_2_ content in plants [38]. Expression of *APX* genes in foliar tissues of higher plants was found to be regulated by unfavorable environmental stimuli, such as wounding, drought, salinity, low and high temperature stresses, heavy metal exposure, exogenous application of H_2_O_2_, NaHS, ABA, oxyfluorfen, ethephon, methyl viologen as well as pathogen infection [2,16,39].

Results of the first stage of experiments evidenced a higher number of aphid-regulated *APX* genes in the seedlings of more resistant Ambrozja variety, compared to susceptible Tasty Sweet genotype. Furthermore, earlier and more profound transcriptional responses of these genes in tissues of Ambrozja cultivar in respect to Tasty Sweet plants were observed. In addition, comparative analyses conducted in the second stage of the biotests revealed greater elevations in *APX1* gene expression in the seedlings of more resistant maize genotypes than in the susceptible ones. Importantly, after 24-h aphids’ colonization, expression levels of the seven *APX* genes in the seedlings of all tested maize varieties were comparable to those in the respective control plants. Additionally, it has been identified two peaks in APX activity (at 8 and 48–96 hpi) in insect-infested seedlings of Ambrozja and Tasty Sweet plants in relation to the non-stressed control. Conversely, after 24-h feeding of the cereal aphids, significant decreases in APX activity occurred in tissues of highly resistant (cv. Ambrozja) plants, while no changes were recorded in more susceptible (cv. Tasty Sweet) seedlings. According to Sytykiewicz [22], *R. padi* or *S. avenae* aphids stimulated profound and constant increases in the content of H_2_O_2_ in several maize genotypes, reaching the maximal levels at 24 hpi. Furthermore, higher elevations in H_2_O_2_ level were reported in aphid-attacked seedlings of more resistant *Z. mays* varieties (Ambrozja, Waza, Touran and Nana), compared to the susceptible ones (Tasty Sweet and Złota Karłowa).

It should be underlined that there are no available reports assessing impact of the aphids’ infestation on transcriptional responses of *APX* genes encoding various isoforms of ascorbate peroxidase in the host plants. However, at the enzymatic level, He *et al.* [40] elucidated that chrysanthemum (*Dendranthema × grandiflora*) plants (Han6, Jinba and Keiun genotypes) infested with *Macrosiphoniella sanbourni* Gillette (Hemiptera: Aphididae) aphids responded significant increases in total activity of APX throughout the studied periods (0.5–168 hpi) relative to the untreated controls. Additionally, markedly higher APX activity was recorded at almost all time points of M. *sanbourni* infestation in more resistant cultivars in comparison with the sensitive (Jinba) genotype. Furthermore, Moloi and van der Westhuizen [19] established that Russian wheat aphids (*Diuraphis noxia*, Mordvilko) evoked a significant increase in level of APX activity in resistant wheat (cv. Tugela DN) plants. The highest elevation (*ca.* 40%) in APX activity in the insect-challenged wheat leaves occurred at 12 hpi, whereas further extending the aphid exposure time up to 48 hpi was linked to progressively lower increments in the estimated parameter compared to the unstressed control. Łukasik *et al.* [41] revealed a different pattern of changes in total activity of APX in the seedling leaves of winter triticale attacked by *R. padi* or *S. avenae* aphids. These authors demonstrated that activity of the examined biocatalyst increased steadily from 24 to 72 hpi in relation to the uninfested plants. Interestingly, Gomez *et al.* [42] demonstrated that a long-term (6- or 9-day) infestation of cotton (*Gossypium hirsutum* L.) leaves with the cotton aphids (*Aphis gossypii* G.) did not influence significant alternations in total activity of APX relative to the non-stressed control. Moreover, it has been well documented that overexpressed or silenced *APX* mutants of the several plant species displayed circumstantial alternations in their growth and development, ROS homeostasis, signaling networks, efficiency of the antioxidative machinery and/or defensive responses to diverse stressing factors [38,43]. Exemplarily, it has been uncovered that the transgenic lines of *A. thaliana* overexpressing *APX* gene from *Jatropha curcas* L., exhibited an elevated salt tolerance in relation to the WT plants [43]. Similarly, *A. thaliana* transformants overexpressing the two transgenes: *APX* and superoxide dismutase (*SOD*), derived from *Rheum australe* Don and *Potentilla atrosanguinea* Lodd., accordingly, characterized with an enhanced salt stress tolerance, increased expression of the genes involved in biosynthesis of the secondary cell wall and encoding the numerous transcription factors (e.g., C3Hs, MYBs, NACs and WRKY) in comparison with the control plants [44]. Based on the results presented in the current study, it has been recognized that the upregulation of few *APX* genes (especially *APX1* and *APX2*) is presumably interconnected with the enhanced resistance of maize toward the cereal aphids. It is postulated that the increments in expression of these aphid-responsive *APX* genes may affect ROS signaling and homeostasis in the insect-stressed plants. However, molecular mechanisms underlying the transcriptional regulation of *APX* isozyme genes as well as their possible biological consequences to the host plant and the aphids still remain to be elucidated.

### 2.4. Impact of the Cereal Aphids’ Infestation on Abundance of Monodehydroascorbate Reductase (MDHAR) Transcripts and MDHAR Activity in the Maize Plants

**Figure 6:**
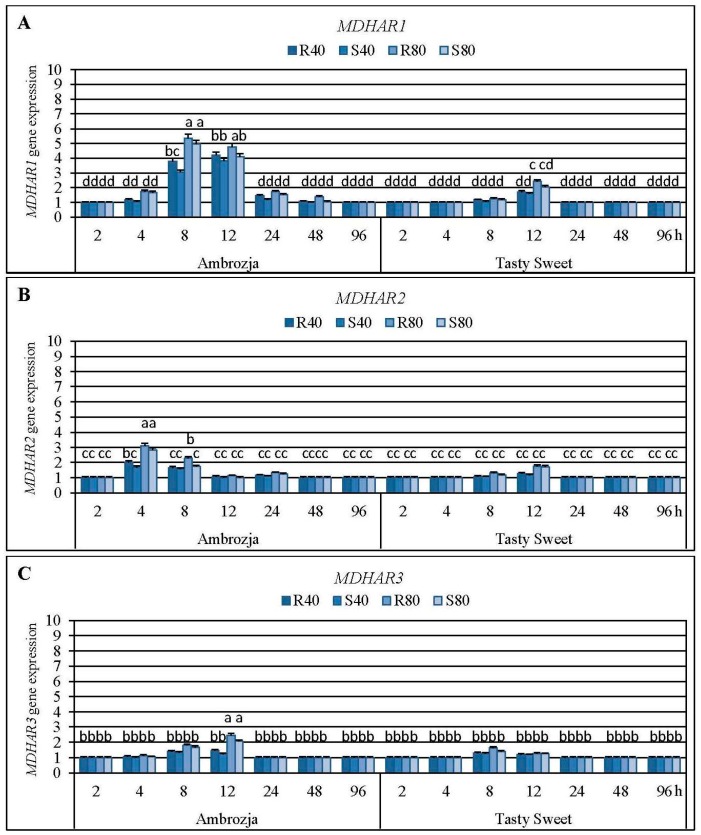
Influence of the cereal aphid’s infestation on relative expression of *MDHAR1* (**A**); *MDHAR2* (**B**) and *MDHAR3* (**C**) genes in the seedlings of Ambrozja and Tasty Sweet maize varieties (first phase of the experiments). R40, R80—maize plants of a certain genotype infested with 40 or 80 *R. padi* females per seedling, accordingly; S40, S80—maize plants of a certain genotype infested with 40 or 80 *S. avenae* females per seedling, respectively. All data are presented as the mean (±SD) of three independent biotests, and significant differences in abundance of the monodehydroascorbate reductase (*MDHAR*) transcripts are denoted by different letters (*p* < 0.05; *post-hoc* Tukey’s test).

Results regarding impact of the tested cereal aphids’ attack on abundance of the four monodehydroascorbate reductase (*MDHAR1*, *MDHAR2*, *MDHAR3* and *MDHAR4*) genes in tissues of Ambrozja and Tasty Sweet maize varieties are depicted in Figure 6, and Supplementary Material, Figure S2. It has been revealed that expression of all the quantified *MDHAR* genes in *Z. mays* seedlings was upregulated in varying degrees in response to *R. padi* or *S. avenae* females’ herbivory. The highest increases were noted in *MDHAR1* mRNA level (7%–435% elevations, depending on a specific variant of the biotests), lower increments occurred in amount of *MDHAR2* and *MDHAR3* transcripts (5%–212% and 3%–146% increases, respectively), while the lowest enhancement (2%–55%) was ascertained in relative expression of *MDHAR4* gene. Time-course analysis revealed that the insect-challenged Ambrozja seedlings responded an accumulation in abundance of *MDHAR1* and *MDHAR4* transcripts from 4 to 48 hpi, *MDHAR2*–4 to 24 hpi, and *MDHAR3*–4 to 12 hpi. In addition, Tasty Sweet plants characterized with increments in expression of all four *MDHAR* genes only at two periods of the aphids’ exposure (*i.e.*, 8 and 12 hpi). The highest increase of *MDHAR1* transcript in Ambrozja seedlings was evidenced at 8 hpi (205%–435% increases), and the maximal elevations (58%–142%) in the target transcript in Tasty Sweet plants were noted at 12 hpi. The largest increases in level of *MDHAR2* gene expression in Ambrozja plants were observed at 4 hpi, while in Tasty Sweet seedlings at 12 hpi (69%–212% and 18%–76% increments, respectively). Importantly, *MDHAR3* and *MDHAR4* genes in Ambrozja plants were maximally upregulated at 12 hpi (24%–145% and 23%–55% increases, correspondingly), whereas the greatest elevations in abundance of these transcripts in Tasty Sweet seedlings occurred at 8 hpi (25%–63% and 10%–31% increments, accordingly). It has also been ascertained that the aphid-infested Ambrozja plants reached higher levels in expression of all four investigated *MDHAR* genes in comparison with Tasty Sweet seedlings. However, Tasty Sweet plants colonized by *R. padi* or *S. avenae* apterae at 12 hpi characterized with higher rises in amount of *MDHAR2* transcript (18%–76% increases in relation to the uninfested control) than Ambrozja seedlings (7%–12% elevations). Furthermore, it has been documented an insect density-dependent changes in expression level of all the studied *MDHAR* genes. Infestation of Ambrozja plants with 80 *R. padi* or *S. avenae* females caused greater increases in abundance of *MDHAR1*, *MDHAR2*, *MDHAR3* and *MDHAR4* transcripts (6%–193%, 5%–112%, 4%–80% and 5%–42% elevations, respectively), compared to the seedlings attacked by 40 aphids of a given species per plant. Moreover, colonization of Tasty Sweet plants by higher number of each of the two studied species of the cereal aphids (80 apterae per seedling) affected 8%–72%, 12%–50%, 6%–33% and 9%–18% higher elevations in expression of the respective *MDHAR* genes in relation to the changes stimulated by 40 insects per plant. It was also evidenced that *R. padi* females possessed a superior effect on accumulation of four examined *MDHAR* transcripts in seedlings of the investigated *Z. mays* genotypes, compared to the grain aphid females. Maize plants infested with the bird cherry-oat aphids reacted higher increments in expression of *MDHAR1*, *MDHAR2*, *MDHAR3* and *MDHAR4* genes (6%–75%, 2%–54%, 5%–39% and 3%–12% increases, accordingly) in relation to *S. avenae*-treated seedlings.

The second phase of the performed experiments unveiled that *MDHAR1* gene expression was upregulated in the seedlings of all six maize cultivars massively infested with the cereal aphids (100 females per plant) (Supplementary Material, Figure S3). The highest transcriptional responses (35%–482% increases) of the target gene in tissues of all the examined *Z. mays* varieties occurred after 12 hpi. At this time point, the strongest elevation in *MDHAR1* mRNA level was recorded in Ambrozja and Waza (highly resistant) seedlings (209%–482% increments, in dependence on the specific treatment), while the lowest increases (35%–90%) were demonstrated in Tasty Sweet and Złota Karłowa (susceptible) plants. Doubling of the insect exposure period (24 hpi) was linked with an upregulation of the target gene expression only in two groups of the maize seedlings (15% and 18% increases in *R. padi*-infested Ambrozja and Waza varieties, respectively). Additionally, at 48 hpi, four groups of the maize plants responded an increase in *MDHAR1* transcript amount (21%, 29%, 14% and 5% increments in *R. padi*-attacked Ambrozja, Waza, Touran and Nana, accordingly) in comparison with the untreated plants. It should be noted that females of bird cherry-oat aphid caused greater increases in *MDHAR1* gene expression in the seedlings of five tested maize genotypes than infestation with *S. avenae* apterae (e.g., 177%, 273%, 110%, 55% and 26% elevations were recorded at 12 hpi in Ambrozja, Waza, Nana, Tasty Sweet and Złota Karłowa cultivars, respectively). In opposite, 12-h colonization of Touran seedlings by *S. avenae* aphids resulted in a 36% higher increment in abundance of *MDHAR1* mRNA in relation to alternations evoked by *R. padi* females.

It has been evidenced an aphid-evoked enhancement in the total activity of MDHAR enzyme in maize seedlings compared to the uninfested controls (Supplementary Material, Figure S4). It has been identified two peaks of the enzyme activity (at 12 and 96 hpi) in the seedlings of both tested maize varieties. Furthermore, earlier and higher stimulation of the MDHAR activity occurred in tissues of Ambrozja variety in relation to Tasty Sweet. Activity of the examined biocatalyst increased at four time points of the cereal aphids’ infestation in Ambrozja plants (8%–15%, 9%–26%, 12%–25% and 23%–35% elevations at 8, 12, 48 and 96 hpi, accordingly) and only after three time intervals in Tasty Sweet plants (5%–11%, 5%–20% and 6%–24% increases at 12, 48 and 96 hpi, respectively). In general, it has been observed a slightly higher (*ca.* 2%–12%) influence of the bird cherry-oat aphids’ infestation on level of MDHAR activity in the maize plants in comparison with *S. avenae* aphids. On the contrary, Ambrozja seedlings colonized by 40 grain aphids per plant (at 48 hpi), and Tasty Sweet seedlings infested with 40 or 80 females of the same insect species (at 48 and 96 hpi, respectively), characterized with 3%–7% higher increments in the enzyme activity relative to *R. padi*-infested plants. 

Factorial ANOVA test corroborated the significant effects of all the examined variables on amount of three *MDHAR* transcripts (*MDHAR1*, *MDHAR2* and *MDHAR3*) in *Z. mays* seedling leaves (Supplementary Material, Tables S5 and S7). All the interactions formulating between the investigated indicators significantly influenced the relative expression of *MDHAR1* gene, almost all of them (9 of 11) markedly affected *MDHAR2* mRNA level, while only two interactions profoundly altered *MDHAR3* transcript amount (Supplementary Material, Table S7). Importantly, changes in expression of *MDHAR4* gene in the maize seedlings were not statistically significant. Furthermore, it was proved that all the tested variables have a significant impact of on the total activity of MDHAR enzyme in *Z. mays* plants; however, the vast majority of the interactions (7 of 11) appeared to be insignificant (Supplementary Material, Table S6).

MDHAR is another enzyme of the AsA-GSH cycle that participates in regeneration of the reduced ascorbate [30]. It catalyses reduction of MDHA radical to AsA with the use of NADPH as an electron donor [15]. In higher plants, MDHAR activity was identified in different cell compartments, such as cytosol, peroxisomes, glyoxysomes, mitochondria and chloroplasts [13,16]. MDHAR isoforms occurring in various plants systems are encoded by multigene families, e.g., *A. thaliana* genome contains five *MDHAR* genes, four *MDHAR* genes were identified in maize, whereas three *MDHAR* transcripts were detected in spinach (*Spinacia oleracea* L.) and tomato (*Lycopersicon esculentum* Mill.) [13,16]. Importantly, it has not yet been clarified which MDHAR isozymes are mainly responsible for maintenance of the optimal intracellular concentration of AsA under physiological and stress conditions. In the current study, the cereal aphids’ herbivory influenced a differential upregulation of expression of the *MDHAR* genes in tested maize plants, depending on the specific maize-aphid treatment. Among the studied transcripts, the highest increment in expression level was demonstrated in case of *MDHAR1* gene. In addition, the seedlings of more resistant maize cultivars (Ambrozja, Waza, Touran and Nana) characterized with markedly higher *MDHAR1* mRNA level (at 12 hpi, second round of the biotests), compared to the susceptible ones (Tasty Sweet and Złota Karłowa). At the enzymatic level, it has been documented aphid-stimulated increases (two peaks at 12 and 96 hpi) in the total activity of MDHAR in seedlings of two tested maize cultivars, however, earlier and stronger elevations were ascertained in more resistant Ambrozja plants in relation to changes in Tasty Sweet plants. Eltelib *et al.* [45] elucidated a circumstantial upregulation of *MDHAR* gene in the leaves of acerola (*Malpighia glabra* L.) in response to salt and low temperature stresses, compared to the untreated controls. Moreover, *M. glabra* plants exposed to dark conditions (8–24 h) reacted a profound gradual decrement in both abundance of the studied transcript and AsA content. According to these authors, the promoter of *MDHAR* gene in the acerola leaves may contain the light-responsive elements. Sultana *et al.* [46] established that the transgenic lines of rice overexpressing *AeMDHAR* gene from *Acanthus ebracteatus* Vahl characterized with an augmented tolerance to salt stress than the untransformed control. It has been also observed considerably higher values of selected yield parameters (such as number of tillers and 1000-grain weight) in the stressed transgenic rice lines in relation to NaCl-treated WT plants, that additionally proved an involvement of MDHAR enzyme in promoting processes of plant growth and development. Brini *et al.* [47], using a high-density microarray technique, demonstrated an upregulation of *MDHAR3* gene in the transgenic *A. thaliana* lines overexpressing wheat *DHN-5* (dehydrin-5) gene. In addition, an elevated AsA accumulation (*ca.* 2.0–2.5-fold) and increased tolerance to oxidative stress in the tested plants in relation to the WT control was recorded. Furthermore, Feng *et al.* [48] ascertained that knockdown of *TaMDHAR* gene in wheat (cv. XZ9104) enhanced its resistance level to *Puccinia striiformis* f. sp. *tritici* (Basidiomycota: Uredinales), a causative agent of the stripe rust. The mutant plants exhibited a lower AsA concentration and APX activity as well as higher H_2_O_2_ content, as compared to the control. Wheat resistance against *P. striiformis* infection was connected with an excessive localized formation of reactive oxygen species in the infected tissues resulting in hypersensitive cell death. Feng *et al.* [49] revealed that wheat (cv. Suwon) plants inoculated with *P. striiformis* race CYR23 responded a repressed expression of *MDHAR4* gene after 12–18 hpi, but prolonged fungal exposure (up to 48 hpi) was associated with significant upregulation of the target gene. It should be emphasized that there are no available reports evidencing impact of the aphids’ herbivory on transcriptional responses of *MDHAR* genes encoding the relevant isoforms in the colonized plants. In addition, there have been published contradictory and/or inconclusive results regarding evaluation of the influence of few insect species’ infestation on expression level of the selected *MDHAR* genes in the hosts [33,50,51]. For example, Little *et al.* [50] revealed that the large white butterfly, *Pieris brassicae* L. (Lepidoptera: Pieridae) oviposition on *A. thaliana* plants resulted in an enhanced *MDHAR* (At5g03630) gene expression (*ca.* 1.5–2.3-fold increases at 24–72 h after the eggs’ deposition) in relation to the unstressed control. On the contrary, Ralph *et al.* [51] evidenced that Sitka spruce (*Picea sitchensis* Bong.) infested with spruce budworms, *Choristoneura occidentalis* Freeman (Lepidoptera: Tortricidae) or white pine weevils, *Pissodes strobi* Peck (Coleoptera: Curculionidae) responded a downregulation of *MDHAR* gene (sequence similarity to *A. thaliana* At5g03630 transcript) compared to the healthy plants.

### 2.5. Transcriptional Responses of Dehydroascorbate Reductase (DHAR) Genes and Activity of DHAR Enzyme in the Aphid-Stressed Maize Cultivars

**Figure 7:**
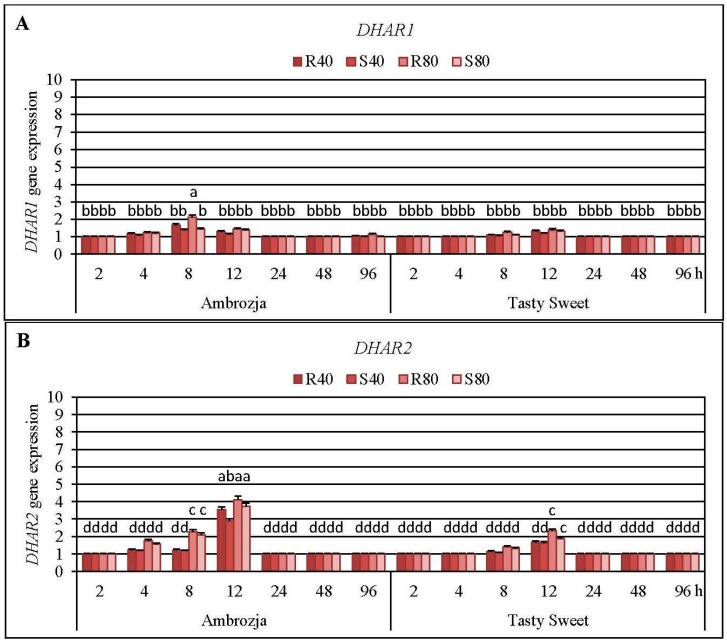
Effect of the cereal aphid’s herbivory on relative expression of *DHAR1* (**A**) and *DHAR2* (**B**) genes in the seedlings of Ambrozja and Tasty Sweet maize varieties (first phase of the experiments). R40, R80—maize plants of a certain genotype infested with 40 or 80 *R. padi* females per seedling, accordingly; S40, S80—maize plants of a certain genotype infested with 40 or 80 *S. avenae* females per seedling, respectively. All data are presented as the mean (±SD) of three independent biotests, and significant differences in abundance of the *DHAR* transcripts are denoted by different letters (*p* < 0.05; *post-hoc* Tukey’s test).

It has been elucidated that *R. padi* or *S. avenae* females’ infestation induced a differential accumulation of all three *DHAR* (*DHAR1*, *DHAR2* and *DHAR3*) transcripts in the maize seedlings of Ambrozja and Tasty Sweet genotypes (Figure 7, and Supplementary Material, Figure S5).

Expression of *DHAR2* gene was highly upregulated (8%–310% elevations, in dependence on the particular experimental variant) in the aphid-infested seedlings of both tested maize varieties, level of *DHAR1* mRNA increased to a much lesser extent (4%–115% increments), whereas amount of *DHAR3* transcript rose in the lowest degree (10%–34% increases). It was found that the cereal aphids’ infestation enhanced abundance of *DHAR1* transcript in Ambrozja plants at four time points (4, 8, 12 and 96 hpi), while amount of the target transcript in Tasty Sweet cultivar increased only at two time intervals of the insect exposure (8 and 12 hpi). The maximal elevation in expression of *DHAR1* gene was recorded at 8 hpi in Ambrozja plants, and after 12 hpi in Tasty Sweet seedlings (37%–115% and 18%–40% increases, accordingly). It should be emphasized that expression of *DHAR2* gene in Ambrozja plants was also earlier stimulated (from 4 to 12 hpi) in response to the cereal aphids’ attack, whereas amount of the target transcript in tissues of Tasty Sweet variety was induced only after two time points (8 and 12 hpi). Furthermore, the greatest aphid-evoked accumulation in abundance of *DHAR2* transcript in *Z. mays* seedlings occurred at 12 hpi (189%–310% and 63%–132% increases in Ambrozja and Tasty Sweet plants, respectively). Colonization of Ambrozja plants with the cereal aphids influenced quite low increases in *DHAR3* gene expression (10%–34%), however, abundance of the target transcript in Tasty Sweet plants remained unaffected in respective to the control. In addition, reconfiguration in *DHAR3* gene expression was observed only at two time points (14%–27% and 10%–34% increases at 4 and 8 hpi, respectively). In general, a higher number of the insects (80 females of *R. padi* or *S. avenae* per plant) resulted in greater increments in expression of all three examined *DHAR* genes in the maize plants in comparison with the lower aphid density (40 apterae per seedling). It has also been unveiled that *R. padi*-infested maize seedlings characterized with a higher accumulation of *DHAR1*, *DHAR2* and *DHAR3* transcripts (3%–68%, 8%–54% and 2%–13% increases, respectively), compared to *S. avenae*-challenged plants. 

Results of the second round of the experiments assessing impact of the cereal aphids’ infestation on abundance of *DHAR2* transcript in tissues of six maize cultivars are presented in Supplementary Material, Figure S6. It was found that the tested hemipterans enhanced the accumulation of *DHAR2* mRNA in the maize seedlings only after a 12-h infestation period. The highest elevations in expression of *DHAR2* gene were detected in Ambrozja and Waza plants (147%–315% increases, depending on the experimental variant), lesser increments (90%–127%) were noted in Touran and Nana seedlings, whereas the lowest increases (16%–77%) occurred in Złota Karłowa and Tasty Sweet cultivars. Moreover, it has been elucidated a stronger influence of *R. padi* apterae feeding on amount of the target transcript in the seedlings of five maize cultivars in relation to the changes affected by the grain aphids (168%, 49%, 37%, 52% and 41% greater increments in Ambrozja, Waza, Touran, Tasty Sweet and Złota Karłowa genotypes, correspondingly). However, *S. avenae*-infested Nana plants characterized with a 11% higher increase in *DHAR2* gene expression compared to the seedlings colonized by the bird cherry-oat aphids. 

It has been ascertained that the cereal aphids’ infestation led to 2%–25%, 3%–10% and 7%–18% elevations at 12, 48 and 96 hpi, respectively, in total activity of DHAR enzyme in Tasty Sweet (susceptible) plants (Supplementary Material, Figure S7). Importantly, the aphid-stressed Ambrozja (highly resistant) seedlings responded earlier and greater increments in level of the quantified parameter (12%–22%, 15%–36%, 27%–49%, and 5%–36% increases at 8, 12, 48 and 96 hpi, correspondingly). The maximal enhancement in activity of DHAR enzyme was demonstrated at 48 hpi in foliar tissues of Ambrozja cultivar, and after 12 or 96 hpi (depending on the specific experimental variant) in Tasty Sweet seedlings. In general, it has been revealed 3%–19% higher increments in level of DHAR activity in *R. padi*-challenged maize plants in comparison with the grain aphids. Conversely, Ambrozja plants colonized by 40 or 80 grain aphids per seedling (at 96 hpi), and Tasty Sweet plants attacked by the lower number of *S. avenae* females (at 48 hpi), possessed 2%–8% higher activity of DHAR in comparison with the maize plants infested with the bird cherry-oat aphids.Factorial ANOVA analysis proved that the examined variables and the interactions significantly influenced expression of *DHAR2* gene in the maize plants (Supplementary Material, Tables S5 and S8). Furthermore, significant effect of two studied parameters (maize genotype and aphid abundance) on amount of *DHAR1* mRNA in the investigated maize seedlings has been confirmed. However, two other tested factors (aphid species and infestation time) and all the interactions did not influence significantly the accumulation of *DHAR1* transcript in *Z. mays* seedlings (Supplementary Material, Table S8). Similarly, slight increases in relative expression of *DHAR3* gene in the maize plants were found to be insignificant. Despite significant influence of all the tested variables on the total activity of DHAR enzyme in maize seedlings was proven, three of eleven interactions did not affect markedly the tested parameter (Supplementary Material, Table S6).

DHAR is the most important biocatalyst in the AsA-GSH cycle, processing the GSH-dependent reduction of DHA in order to maintain the sufficient level of AsA and ascorbate redox state in plant tissues [30]. In maize genome, three *DHAR* genes (*DHAR1*, *DHAR2* and *DHAR3*) encoding the relevant DHAR isoforms were also identified [16]. Additionally, it has been reported that *A. thaliana* genome contains five *DHAR*-like isoform genes, however, only three of the genes are being transcribed to the fully functional isoenzymes (mitochondrial DHAR1, cytosolic DHAR2 and chloroplastic DHAR3) [13,52]. It has been evidenced that alternations in DHAR activity profoundly affected intensity of AsA recycling in plant systems, thus influencing their growth, development and aging processes [53]. Elevations in total activity of DHAR have been recorded in tissues of the several plants subjected to abiotic stress conditions (e.g., drought, low temperature, salinity, osmotic stress, salicylic acid treatment and heavy metals’ exposure) [15,53]. Huang and Song [54] evidenced that dried embryos of maize (cv. Nongda 108) exhibited an increased total activity of DHAR in relation to the fresh ones. In contrast, *Z. mays* embryos excised from the germinating maize seeds treated with −0.6 and −1.2 MPa polyethylene glycol solutions characterized with declined levels of DHAR activity in relation to the unstressed control, thus evidencing a profound repression of AsA regeneration. Talaat [55] revealed that exogenous application of effective microorganisms (EM) suspension resulted in a significantly higher activity of DHAR in leaves of the common bean (cv. Nebraska) subjected to saline conditions in relation to those untreated with EM. Chen *et al.* [56] ascertained that leaves of the transgenic maize plants overexpressing the wheat *DHAR* gene displayed remarkably enhanced DHAR activity (up to 50-fold increases) and elevated AsA contents (*ca.* 2-fold) in respective to the WT control. Additionally, it has been observed an increment (up to 40%) in foliar ascorbate redox state in *DHAR*-overexpressing maize lines compared to the untransformed control. It provided a strong evidence indicating the pivotal role of DHAR enzyme in restoring the AsA pool in *Z. mays* leaves. It has also been increasingly reported that overexpression of *DHAR* genes in numerous plant systems conferred an elevated tolerance toward a broad range of detrimental environmental stimuli (e.g., herbicide treatment, heavy metals’ exposure, drought, cold, oxidative or salt stress) [13,16,57]. However, regulation of transcriptional responses of *DHAR* genes is highly complex and differed markedly between the tested model plants and nature of the stressing factors [58,59]. Until now, there are no available reports assessing influence of aphids’ infestation on transcriptional responses of the genes encoding the compartment-specific DHAR isoforms in tissues of any mono- or dicotyledonous plant species. However, at the biochemical level, Kerchev *et al.* [11] demonstrated that the leaves of potato (cv. Desiree) plants infested with the green peach aphids did not respond any significant disturbances in total activity of DHAR enzyme (*i.e.*, slight increase at 8 hpi, and narrow decreases at 24 and 48 hpi) compared to the unstressed control. Bodenhausen and Reymond [59] revealed that *A. thaliana* ecotype Col-0 plants infested with the small cabbage white butterfly, *Pieris rapae* L. (Lepidoptera: Pieridae) or Egyptian cotton worm responded a significant upregulation of *DHAR1* gene (at 5 hpi, 5.1- and 4.5-fold increases, respectively), compared to the unstressed control. Importantly, *coi1-1* (*coronatine insensitive 1*) mutant of *A. thaliana* plants colonized by *P. rapae* or *S. littoralis* did not respond any significant alternations in abundance of *DHAR1* transcript. It provided an indirect evidence that jasmonate pathway signaling is a crucial factor in transcriptional upregulation of *DHAR1* gene in the thale cress plants infested with the examined lepidopterans.

### 2.6. Insect-Induced Changes in Relative Expression of Glutathione Reductase (GR) Genes and GR Activity in Z. mays Seedlings

**Figure 8:**
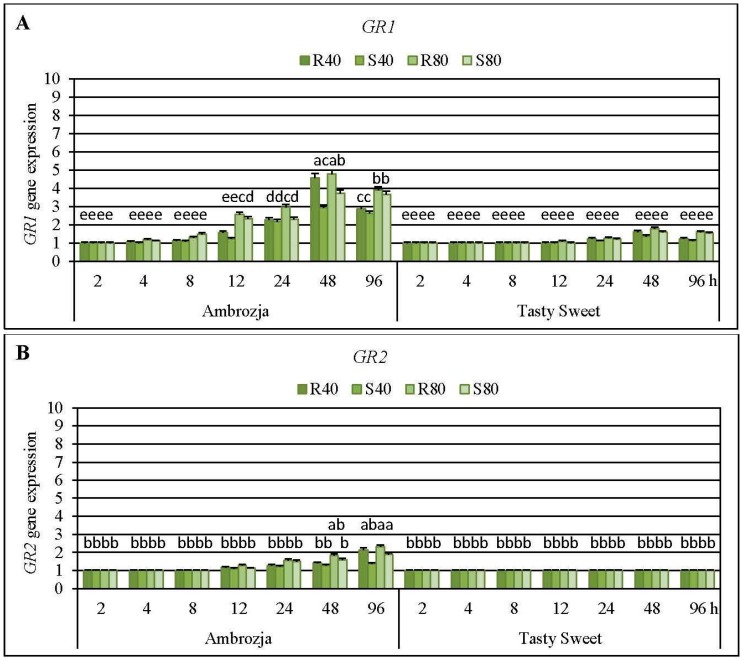
Influence of the cereal aphid’s attack on relative expression of *GR1* (**A**) and *GR2* (**B**) genes in the seedlings of Ambrozja and Tasty Sweet maize varieties (first phase of the experiments). R40, R80–maize plants of a certain genotype infested with 40 or 80 *R. padi* females per seedling, accordingly; S40, S80–maize plants of a certain genotype infested with 40 or 80 *S. avenae* females per seedling, respectively. All data are presented as the mean (±SD) of three independent biotests, and significant differences in abundance of the *GR* transcripts are denoted by different letters (*p* < 0.05; *post-hoc* Tukey’s test).

Short-time (2-h) infestation of Ambrozja or Tasty Sweet seedlings with the tested cereal aphids (*R. padi* or *S. avenae*) did not affect modulations in expression of two examined GR (*GR1* and *GR2*) genes (Figure 8). Level of *GR1* mRNA in the insect-stressed Ambrozja seedlings raised gradually from 4 to 48 hpi (6%–19% to 195%–378% increments, accordingly), but longer exposure to the hemipterans’ feeding (96 hpi) caused a lower increase in expression of the target gene (162%–291% elevations). In addition, delayed and insignificant upregulation of *GR1* gene was demonstrated in the insect-challenged Tasty Sweet plants (10%–26% elevations at 24 hpi, 38%–79% at 48 hpi, 12%–60% at 96 hpi). Importantly, Ambrozja seedlings infested with the cereal aphids displayed higher increments in abundance of *GR1* transcript (8%–378% increases) in relation to Tasty Sweet genotype (6%–43% increases). Moreover, amount of *GR2* transcript in Ambrozja plants increased steadily from 12 to 96 hpi (14%–25% to 36%–128% elevations, respectively), compared to the relevant uninfested controls. However, all the tested insect treatments did not influence the abundance of *GR2* transcript in Tasty Sweet seedlings. Furthermore, *R. padi* apterae affected greater increments in relative expression of *GR1* or *GR2* genes in seedlings of the tested maize cultivars than the grain aphid females (e.g., 80 *R. padi* females influenced 9%–103% and 5%–19% higher increases in level of *GR1* mRNA in Ambrozja and Tasty Sweet varieties, accordingly, compared to changes evoked by the equal number of the grain aphid females). The only exception was the expression of *GR1* gene in Ambrozja seedlings infested with 80 *S. avenae* females at 8 hpi that possessed a 22% higher amount of the target transcript in relation to the respective plants attacked by *R. padi*. An insect density-dependent scale of alternations in abundance of the analyzed *GR* transcripts was also revealed―higher number of the hemipterans (80 apterae per seedling) evoked 4%–116% and 7%–53% greater increases in abundance of *GR1* and *GR2* transcripts, respectively, than lower level of the insects’ density (40 females per plant). 

The second phase of the biotests revealed that the cereal aphids’ infestation resulted in a differential upregulation of *GR1* gene in the seedlings of all six investigated maize genotypes (5%–207%, 7%–239% and 14%–422% elevations recorded at 12, 24 and 48 hpi, respectively) (Supplementary Material, Figure S6). The highest increments in expression of the target gene were noted after 48 hpi in Ambrozja and Waza varieties (236%–308% and 294%–420% increases, correspondingly), whereas the lowest increases were observed in *S. avenae*-colonized seedlings of Tasty Sweet (5% increase at 12 hpi) and Złota Karłowa (7% increase at 24 hpi). Furthermore, infestation with the bird cherry-oat aphids provoked more circumstantial elevations in *GR1* mRNA level (20%–68%, 2%–131% and 11%–126% higher increments at 12, 24 and 48 hpi, accordingly) in tissues of all six tested *Z. mays* genotypes in respect to the changes induced by *S. avenae* aphids.

Infestation of the maize seedlings with the cereal aphids caused delayed increases (at 12–96 hpi and 24–96 hpi in Ambrozja and Tasty Sweet plants, respectively) in the total activity of GR enzyme, compared to the respective controls (Supplementary Material, Figure S7). However, earlier and stronger increases in the GR activity occurred in the insect-attacked Ambrozja (resistant) plants relative to Tasty Sweet (susceptible) cultivar. The highest rise in activity of the examined biocatalyst in foliar tissues of both investigated *Z. mays* genotypes was noted at 48 hpi (113%–140% and 46%–63% elevations in Ambrozja and Tasty Sweet plants, accordingly). In addition, evidence has shown a more substantial effect of the bird cherry-oat aphids on level of GR activity (*i.e.*, 4%–25% greater increases in values of the measured parameter) in the maize seedlings in relation to changes evoked by *S. avenae* apterae. However, four exceptions were recorded; Ambrozja plants infested with 40 grain aphids per plant (at 12 and 96 hpi), seedlings of the same cultivar attacked by 80 grain aphids per plant (at 96 hpi), and Tasty Sweet seedlings colonized by the lower number of grain aphids (at 48 hpi), possessed 7%–19% higher increments in GR activity than respective groups of *R. padi*-infested maize plants.

The factorial ANOVA test confirmed significant impact of all the investigated indicators and their interrelationships on abundance of *GR1* transcript in *Z. mays* seedlings (Supplementary Material, Tables S5 and S9). Additionally, all the examined factors (maize genotype, aphid species, aphid abundance and infestation time), and most of the studied interactions (six of eleven), significantly affected expression of *GR2* gene (Supplementary Material, Table S9). Moreover, all the tested variables influenced the total activity of GR enzyme in maize plants, however, five interactions were not significant (Supplementary Material, Table S6).

GRs comprise a group of flavoprotein oxidoreductases responsible for NADPH-dependent regeneration of the reduced glutathione, and thus participate in maintaining both adequate GSH/GSSG ratio and high efficiency of the AsA-GSH cycle [32,53]. Subcellular fractionation of the tissue homogenates derived from numerous plant species revealed the presence of a few GR isoforms in the several cell compartments, such as cytosol, chloroplasts, peroxisomes and mitochondria [13,16]. Three *GR* transcripts were identified in rice and wheat, whereas two *GR* genes were found in genomes of maize and barley [16]. There has been increasingly reported that total activity of GR in maize tissues was profoundly regulated during developmental processes as well as under exposure to multitude environmental stimuli [60,61]. Durmuş and Bekircan [61] established that exposure to diuron (herbicide repressing the photosystem II) boosted the total activity of GR in the maize leaves. In addition, pre-treatment of *Z. mays* plants with putrescine caused an increment in GR activity, and additionally, it counteracted the diuron-induced increase in activity of superoxide dismutase. Similarly, Kocsy *et al.* [60] proved that the maize seedlings (Z7 and Penjalinan genotypes) subjected to osmotic, low or high temperature stresses responded significant elevations in the total activity of GR, compared to the non-stressed control. Recently, Ding *et al.* [62] documented that the transgenic *A. thaliana* plants with downregulated *GR2* gene characterized with an earlier onset of the leaf senescence process, compared to the WT plants. Furthermore, transcriptomic analyses demonstrated an upregulation of the numerous genes associated with biogenesis of phytohormones, oxidative stress and senescence in these mutant plants. Additionally, Li *et al.* [3] ascertained that the flag leaves of early senescence leaf (*esl*) mutant of rice displayed a higher activity of GR up to 21 days post anthesis relative to the WT plants. According to these authors, the elevated expression of *GR1* and *GR3* genes were recorded at early phase of senescence, while abundance of *GR2* mRNA declined progressively during the investigated period in the *esl* plants, compared to the wild-type ones. In this report, it has been demonstrated that the aphid-stimulated upregulation in expression of *GR1* and *GR2* genes, in parallel with the enhancement in GR activity in the leaves of *Z. mays* seedlings. Detailed analyses revealed an earlier and higher accumulation of *GR1* mRNA in the insect-treated plants in comparison with *GR2* transcript. Additionally, aphid-stressed seedlings of more resistant maize varieties responded greater upregulation of *GR1* gene in respect to the susceptible ones. Furthermore, cereal aphids’ infestation caused substantially higher increases in level of GR activity (at 48–96 hpi) in the seedlings of highly resistant Ambrozja variety than in the foliar tissues of susceptible Tasty Sweet plants. Presumably, the accelerated regeneration of the reduced glutathione resulted in increments in GSH content and GSH/GSSG ratio in Ambrozja seedlings. Therefore, it is postulated the crucial significance of the reduced glutathione pool and/or the GSH/GSSG ratio in conferring enhanced maize resistance toward the tested hemipterans. It is important to underline the lack of available reports evaluating the insect-evoked transcriptional responses of *GR* genes encoding the respective isozymes present in various subcellular compartments in the host plants. However, at the biochemical level, Gomez *et al.* [42] revealed a significant increment in total activity of GR in the leaves of cotton plants after 8-day feeding of the cotton aphids in respect to the uninfested control. Moreover, Moloi and van der Westhuizen [19] ascertained that resistant *T. aestivum* (cv. Tugela DN) plants infested with the Russian wheat aphids reacted an elevation in the total activity of GR, with the highest increase recorded at 12 hpi. After this period, there have been demonstrated progressively lower increments in the enzyme activity compared to the control. Importantly, only slight and insignificant alternations in level of GR activity in tissues of susceptible wheat (cv. Tugela) under the insect attack were demonstrated by these authors. Conversely, Kerchev *et al.* [11] did not reveal any significant alternations in GR activity (*i.e.*, slight decrease at 8 hpi, and a slight gradual increment at 24 and 48 hpi) in *S. tuberosum* leaves colonized by the green peach aphid females.

## 3. Experimental Section

### 3.1. Plants

Six maize genotypes differing with susceptibility levels to *R. padi* and *S. avenae* infestation were included in the biotests: Ambrozja and Waza (highly resistant), Nana and Touran (moderately resistant), Tasty Sweet and Złota Karłowa (susceptible) [21]. The maize seeds were purchased from the local grain companies: PNOS S.A. (Ożarów Mazowiecki, Poland) and Reheza (Moszna, Poland). Seeds were subjected to surface sterilization, according to the procedure described by Sytykiewicz [21]. Maize seedlings were individually planted in round plastic pots (10 cm of diameter × 9 cm in height) with the universal soil, and without any supplemental fertilization. The plants were cultivated in a climate controlled chamber under a long-day photoperiod (LD; 16 h light, 8 h dark), at 22 °C ± 2 °C/16 °C ± 2 °C (day/night), light intensity of 100 μM·m^−2^·s^−1^, and relative humidity of 65% ± 5%. 

### 3.2. Aphids

Adult apterous parthenogenetic females of two investigated cereal aphids’ species (*R. padi* and *S. avenae*) were sampled from cereal crops within the Siedlce district in Poland (52°09′54″N, 22°16′17″E). Subsequently, the aphids’ specimens were transferred to the laboratory of the Department of Biochemistry and Molecular Biology (University of Natural Sciences and Humanities in Siedlce, Poland). The gathered hemipterans formed the relevant mother stocks on the seedlings of common wheat (*Triticum aestivum* L., cv. Tonacja), and the aphids settled down on new wheat plants provided every week. Aphid stocks were maintained for a year in a climate controlled chamber, under the conditions specified in the Section 3.1. The leaf infestation experiments were carried out with the use of wingless parthenogenetic females originated from the relevant stock cultures.

### 3.3. Leaf Infestation Procedure

In order to ensure complete randomization of the study, three independent series of the biotests were carried out. The first phase of the experiments was performed on 14-day-old maize seedlings of both examined genotypes (Ambrozja and Tasty Sweet) that were artificially infested with adult wingless females of *R. padi* or *S. avenae*. Two levels of leaf infestation were tested: 40 or 80 insects of a given species per plant (8 or 16 aphids per leaf). Additionally, groups of the control seedlings (*n* = 20) were uninfested with the hemipterans. It should be noted that only healthy plants of the comparable height were chosen for conducting the bioassays. Insect-colonized and untreated (control) seedlings were caged separately in gauze-covered plastic cylinders (20 cm of diameter × 50 cm in height). After 2, 4, 8, 12, 24, 48 and 96 h post initial aphid infestation (hpi), the insects were gently removed from the colonized plants, and next, the leaves from both aphid-infested and control seedlings were cut off, and immediately subjected for further analytical procedures (*i.e.*, gene expression quantification, determination of total activity of AsA-GSH cycle enzymes, and measurement of GSH, GSSG, AsA and DHA contents).

The second stage of the biotests was performed on 14-day-old seedlings of six maize varieties displaying different susceptibility degrees to the cereal aphids’ infestation: Ambrozja, Waza, Nana, Touran, Tasty Sweet and Złota Karłowa. Each tested group of plants (20 seedlings per single treatment) was artificially infested with adult apteral parthenogenetic females of *R. padi* or *S. avenae* (100 aphids per seedling; 20 aphids per leaf). Control plants of each genotype (*n* = 20) were uninfested with the insects. Subsequently, aphid-colonized and aphid-free maize seedlings were isolated with plastic cylinders, as described above. Relative expression of the analyzed genes and content of GSH, GSSG, AsA and DHA in foliar tissues of *Z. mays* plants were determined at three selected time points (12, 24 and 48 hpi).

### 3.4. Determination of Glutathione Content 

Extraction of the glutathione from maize seedlings was performed following the procedure described by Su *et al.* [63]. The freshly collected seedling leaves (portions of 100 mg each) were homogenized in liquid nitrogen, and next, 400 mm^3^ of *Glutathione Reaction Buffer* (*GRB*) and 100 mm^3^ of 5% sulfosalicylic acid (SSA) were added. After a brief and vigorous vortexing, the probes were incubated on ice for 5 min, and centrifuged at 10,000×·*g* for 10 min (at 4 °C). The supernatants were transferred to Eppendorf tubes, and protein impurities were removed from the solution by using 10 kDa MW cut-off spin filter. Subsequently, total and reduced glutathione content in the tested samples was measured with the use of *ApoGSH^TM^ Glutathione Colorimetric Detection Kit* (BioVision Inc., Milpitas, CA, USA), following the manufacturer’s protocol. Absorbance of the probes was recorded at 405 nm (*A*_405_) in 96-well and flat-bottom microplates, using an Epoch UV-Vis spectrophotometer (BioTek, Winooski, VT, USA). The content of total and reduced glutathione in the samples were calculated from the calibration curve prepared from the lyophilized *GSH Standard* (BioVision Inc., Milpitas, CA, USA), according to the manufacturer’s instructions. Content of oxidized glutathione was calculated as the difference between total and reduced glutathione. The amount of GSH or GSSG in *Z. mays* plants was expressed in nmol per gram of fresh weight (FW). Furthermore, the redox ratio of GSH/GSSG was determined.

### 3.5. Determination of Ascorbate Amount

Fresh leaf samples (500 mg) were ground in liquid nitrogen, and thereafter, the fine powder was homogenized in 2 cm^3^ of 6% trichloroacetic acid (TCA), and the mixture was centrifuged at 13,000×·*g* for 10 min (at 4 °C). The obtained supernatant was subjected to quantification of the ascorbate. Total and reduced ascorbate contents in maize seedling leaves were determined according to the protocol described by Gillespie and Ainsworth [64]. Absorbance of the samples was measured at 525 nm (*A*_525_) in 96-well and flat-bottom microplates, using an Epoch UV-Vis spectrophotometer (BioTek). The amount of total and reduced ascorbate was calculated from the calibration curve prepared with the standard l-ascorbic acid (Sigma-Aldrich, Poznań, Poland). Concentration of the oxidized ascorbate (DHA) was calculated as the difference between total and reduced ascorbate. The amount of foliar AsA or DHA in the maize seedlings was expressed in nmol per gram of fresh weight. In addition, the redox ratio of AsA/DHA was estimated. 

### 3.6. Total RNA Isolation and cDNA Synthesis

Leaves harvested from aphid-stressed and uninfested maize seedlings were immediately frozen in liquid nitrogen; thereafter, foliar tissues were ground to a fine powder. Total RNA was extracted with the use of *Spectrum Plant Total RNA Kit* (Sigma-Aldrich). Residual genomic DNA was hydrolysed with *On-Column DNase I Digestion Set* (Sigma-Aldrich). Determination of RNA concentration and evaluation of its purity was performed by using an Epoch UV-Vis microplate spectrophotometer (BioTek). Only intact RNA preparates exhibiting the high integrity (*A*_260/280_ > 2.0, and *A*_260/230_ > 1.8) were used for the reverse transcription under *in vitro* conditions. First-strand cDNA synthesis was accomplished with *High Capacity cDNA Reverse Transcription Kit with RNase Inhibitor Kit* (Life Technologies, Warsaw, Poland). Moreover, two negative controls: no reverse transcriptase (NRT) and no template (NTC) were provided during each round of the reactions. RNA and cDNA preparates were stored at −80 and −20 °C, respectively.

### 3.7. Measurement of Relative Gene Expression

Quantification of the target mRNA abundance in the seedling leaves of maize was performed with application of real-time qRT-PCR technique. Glyceraldehyde-3-phosphate dehydrogenase (*GAPDH*) gene (*GenBank* accession no. Pr032251180) was selected as an internal reference. The relative expression of all seven *APX* genes (*APX1*, *APX2*, *APX3*, *APX4*, *APX5*, *APX6* and *APX7*) in maize seedlings was measured using *TaqMan Gene Expression Assays* (Life Technologies). The list of *GenBank* reference sequences and identification numbers of the gene-specific assays for all seven *APX* genes are depicted in Supplementary Material (Table S10). Expression levels of the other studied *Z. mays* genes (*MDHAR1*, *MDHAR2*, *MDHAR3*, *MDHAR4*, *DHAR1*, *DHAR2*, *DHAR3*, *GR1* and *GR2*) were evaluated with the use of *Custom TaqMan Gene Expression Assays* (Life Technologies). Sequences of primers and fluorescent probes designed for *MDHAR*, *DHAR* and *GR* genes are shown in Supplementary Material (Tables S11 and S12). Amplification of DNA was monitored using fast mode of *StepOne Plus Real-Time PCR System*, equipped with the *StepOnePlus Software v2.3* (Applied Biosystems, USA). Thermal cycling conditions were programmed as previously described [21]. Relative expression of the tested genes was assayed in *MicroAmp Fast Optical* 96-well reaction plates (Life Technologies), and the reaction mixtures comprised 2× *TaqMan Fast Universal PCR Master Mix* (10 mm^3^), 20× *TaqMan Gene Expression* *Assay* (1 mm^3^), cDNA (4 mm^3^), and RNase-free water (5 mm^3^). Expression data of each investigated gene were analyzed using the comparative *C*_t_ (ΔΔ*C*_t_) method of Livak and Schmittgen [65]. The results were displayed as *n*-fold changes (±SD) in the examined mRNA level in the insect-stressed plants in relation to the uninfested control. Four biological and three technical replications were provided for each set of qRT-PCR assays.

### 3.8. Extraction Procedure and Assays of AsA-GSH Cycle Enzymes’ Activity

Freshly collected maize seedling leaves were immediately ground in liquid nitrogen, and the obtained powder was stored at −80 °C. Next, approx. 0.50 g of each sample was homogenised in extraction solution (50 mM sodium phosphate buffer, pH 7.8, containing 1% (*w*/*v*) of polyvinylpyrrolidone, 1 mM ethylenediaminetetraacetic acid (EDTA) and 5 mM MgCl_2_ [2]. Extraction medium for assaying of APX activity contained additionally 2 mM of AsA. The homogenate was filtered through four layers of nylon cloth and centrifuged at 15,000×·*g* for 20 min at 4 °C. The supernatant was decanted, stored at −80 °C, and used for subsequent determination of total activity of AsA-GSH cycle enzymes (*i.e.*, APX, MDHAR, DHAR and GR). The total activity of all the examined enzymes was determined at 25 °C.

Activity of APX in *Z. mays* foliar tissues was estimated according to the procedure developed by Nakano and Asada [66]. The reaction mixture (1 cm^3^) contained 50 mM sodium phosphate buffer (pH 7.0), 0.5 mM AsA, 0.1 mM H_2_O_2_ and 50 mm^3^ of the sample. Hydrogen peroxide-dependent decline in reduced ascorbate was monitored for 5 min at 30-s intervals (ε = 2.8 mM^−1^·cm^−1^). Absorbance value of the mixture was measured at 290 nm using an Epoch microplate UV-Vis spectrophotometer (BioTek). Activity of APX was expressed in nanomoles of ascorbate oxidized per minute per milligram of protein. 

Activity of MDHAR was assayed as described by Liu *et al.* [16], with slight modifications. MDA was generated by AsA/ascorbate oxidase system. The reaction mixture (1 cm^3^) contained 50 mM sodium phosphate buffer (pH 7.5), 2 mM AsA, 1 U of ascorbate oxidase, 0.2 mM NADH, and 50 mm^3^ of the sample. Oxidation rate of NADH was monitored for 3 min, by recording changes in the absorbance value at 340 nm (ε = 6.22 M^−1^·cm^−1^). Activity of MDHAR was expressed in nanomoles of NADH oxidized per minute per milligram of protein.

Activity of DHAR was assessed with the use of Hossain and Asada method [67]. The reaction medium (1 cm^3^) consisted of 50 mM sodium phosphate buffer (pH 7.0), 2.5 mM GSH, 0.5 mM DHA and 50 mm^3^ of the sample. Glutathione (GSH)-dependent formation of AsA was monitored for 3 min, and an increase in the absorbance level was recorded at 265 nm (ε = 14 mM^−1^·cm^−1^). Activity of DHAR was expressed in nanomoles of AsA formed per minute per milligram of protein. 

Activity of GR was determined using the method described by Donahue *et al.* [68], with slight modifications. The reaction solution (1 cm^3^) consisted of 50 mM sodium phosphate buffer (pH 7.0), 1 mM GSSG, 1 mM EDTA, 5 mM MgCl_2_, 0.2 mM NADPH and 50 mm^3^ of the sample. Glutathione (GSSG)-dependent oxidation of NADPH was monitored for 2 min, and decrease in the absorbance value was measured spectrophotometrically at 340 nm (ε = 6.22 M^−1^·cm^−1^). Activity of GR was expressed in nanomoles of NADPH oxidized per minute per milligram of protein.

Protein determination in each foliar extract was carried out using Lowry method [69]. Bovine serum albumin was used as the standard for preparation of calibration curve.

### 3.9. Statistics

All data are expressed as the average (±SD) of at least three independent replicates. In order to assess the significance of the influence of four investigated variables (maize genotype, aphid species, insect density and infestation time) and their interactions, the factorial analysis of variance (ANOVA) was employed. Subsequently, *post-hoc* Tukey’s test was performed, and the significance level was set at *p* < 0.05. Statistical analyses were conducted with application of the STATISTICA 10 software (StatSoft, Kraków, Poland).

## 4. Conclusions

The time-course analysis unveiled dissimilar patterns of the aphid-stimulated changes in expression profiles of the AsA-GSH cycle-related genes, content of the analyzed low-molecular antioxidants (*i.e.*, AsA and GSH) and their redox ratios in the seedling leaves of maize cultivars, displaying different resistance levels toward the tested hemipterans (*R. padi* or *S. avenae* apterae). Collectively, the cereal aphids’ infestation evoked greater reconfigurations in transcriptional responses of the several studied genes in foliar tissues of more resistant *Z. mays* varieties in comparison with the susceptible ones. Importantly, differential modulations in the total activity of APX, MDHAR, DHAR and GR enzymes were documented, as well as earlier and substantially higher alternations in the amount of oxidized and reduced forms of glutathione and ascorbate in the seedling leaves of highly resistant Ambrozja variety, compared to more susceptible Tasty Sweet plants. The pivotal importance of the reduced glutathione amount and/or the GSH/GSSG ratio in conferring the enhanced resistance of maize seedlings against the tested insects wasproved. The presented results also evidenced a more substantial impact of oligophagous *R. padi* herbivory on expression of the numerous AsA-GSH cycle-related isozyme genes, and other quantified parameters in seedlings of the investigated maize cultivars, relative to changes triggered by monophagous *S. avenae* aphids. Moreover, it has been highlighted the insect-density effect on scale of the changes in levels of the quantified parameters in the maize plants. Importantly, it has been identified a wide array of marker genes circumstantially upregulated in the aphid-challenged seedlings of more resistant maize genotypes. Among them, the four transcripts (*i.e.*, *APX1*, *MDHAR1*, *DHAR2* and *GR1*) were remarkably accumulated in tissues of less susceptible *Z. mays* varieties. Hence, it is highly probable that these genes may contribute to increasing resistance of the maize genotypes toward the examined hemipterans. On the one hand, quantification of the relative expression of the aphid-responsive genes may be utilized as a molecular tool in screening of *Z. mays* cultivars for resistance to the cereal aphids’ attack, and additionally, they may be subjected to advanced biotechnological approaches in order to improve plant tolerance to the hemipterans’ infestation, on the other hand. However, further in-depth studies are needed to assess influence of *R. padi* or *S. avenae* apterae colonization on genome-wide transcriptomic reconfigurations (at both mRNA and microRNA levels) in the maize cultivars, differing in resistance degrees to the cereal aphids.

## Supplementary Materials

Supplementary materials can be found at http://www.mdpi.com/1422-0067/17/3/268/s1.
