# Supplementary material for: Expression Patterns of Genes Involved in Ascorbate-Glutathione Cycle in Aphid-Infested Maize (Zea mays L.) Seedlings
Source: Int J Mol Sci. 2016 Feb 23;17(3):268. doi: 10.3390/ijms17030268 (PMC4813132; doi:10.3390/ijms17030268)
Supplement: Supplementary file 1 [file ijms-17-00268-s001.pdf]

Supplementary Materials: Expression Patterns of Genes Involved in Ascorbate-Glutathione Cycle in Aphid-Infested Maize (*Zea mays* L.) Seedlings

Hubert Sytykiewicz

Table S1. Factorial ANOVA of the four examined variables (maize genotype, aphid species, aphid abundance, infestation time), and their interrelations on concentration of reduced glutathione (GSH), oxidized glutathione (GSSG), reduced ascorbate (AsA) and oxidized ascorbate (DHA) in the seedling leaves of maize.

Tested Factors and Interactions	GSH	GSSG	AsA	DHA
Maize genotype (G)	$F_{1, 192} = 3499.5$ ($p < 0.001$)	$F_{1, 192} = 3124.6$ ($p < 0.001$)	$F_{1, 192} = 1025.4$ ($p < 0.001$)	$F_{1, 192} = 9152.0$ ($p < 0.001$)
Aphid species (S)	$F_{2, 192} = 437.0$ ($p < 0.001$)	$F_{2, 192} = 1581.1$ ($p < 0.001$)	$F_{2, 192} = 116.7$ ($p < 0.001$)	$F_{2, 192} = 1706.8$ ($p < 0.001$)
Aphid abundance (A)	$F_{1, 192} = 2058.2$ ($p < 0.001$)	$F_{1, 192} = 182.4$ ($p < 0.001$)	$F_{1, 192} = 868.2$ ($p < 0.001$)	$F_{1, 192} = 1895.4$ ($p < 0.001$)
Infestation time (T)	$F_{7, 192} = 6780.5$ ($p < 0.001$)	$F_{7, 192} = 1415.2$ ($p < 0.001$)	$F_{7, 192} = 6104.3$ ($p < 0.001$)	$F_{7, 192} = 1366.9$ ($p < 0.001$)
G × S	$F_{2, 192} = 34.1$ ($p < 0.001$)	$F_{2, 192} = 489.9$ ($p < 0.001$)	$F_{2, 192} = 820.5$ ($p < 0.001$)	$F_{2, 192} = 1995.0$ ($p < 0.001$)
S × A	$F_{2, 192} = 514.9$ ($p < 0.001$)	$F_{2, 192} = 140.5$ ($p < 0.001$)	$F_{2, 192} = 279.1$ ($p < 0.001$)	$F_{2, 192} = 528.4$ ($p < 0.001$)
G × A	$F_{1, 192} = 122.4$ ($p < 0.001$)	$F_{1, 192} = 128.1$ ($p < 0.001$)	$F_{1, 192} = 4.9$ ($p = 0.017$)	$F_{1, 192} = 232.5$ ($p < 0.001$)
S × T	$F_{14, 192} = 3191.1$ ($p < 0.001$)	$F_{14, 192} = 240.6$ ($p < 0.001$)	$F_{14, 192} = 1554.2$ ($p < 0.001$)	$F_{14, 192} = 816.1$ ($p < 0.001$)
G × T	$F_{7, 192} = 1537.0$ ($p < 0.001$)	$F_{7, 192} = 918.3$ ($p < 0.001$)	$F_{7, 192} = 506.8$ ($p < 0.001$)	$F_{7, 192} = 762.5$ ($p < 0.001$)
A × T	$F_{7, 192} = 122.8$ ($p < 0.001$)	$F_{7, 192} = 105.2$ ($p < 0.001$)	$F_{7, 192} = 345.1$ ($p < 0.001$)	$F_{7, 192} = 163.9$ ($p < 0.001$)
G × S × A	$F_{2, 192} = 65.4$ ($p < 0.001$)	$F_{2, 192} = 9.6$ ($p = 0.002$)	$F_{2, 192} = 10.0$ ($p = 0.008$)	$F_{2, 192} = 87.2$ ($p < 0.001$)
G × S × T	$F_{14, 192} = 427.8$ ($p < 0.001$)	$F_{14, 192} = 224.5$ ($p < 0.001$)	$F_{14, 192} = 134.2$ ($p < 0.001$)	$F_{14, 192} = 214.6$ ($p < 0.001$)
S × A × T	$F_{14, 192} = 34.1$ ($p < 0.001$)	$F_{14, 192} = 42.0$ ($p < 0.001$)	$F_{14, 192} = 18.3$ ($p < 0.001$)	$F_{14, 192} = 92.5$ ($p < 0.001$)
G × A × T	$F_{7, 192} = 30.5$ ($p < 0.001$)	$F_{7, 192} = 19.4$ ($p < 0.001$)	$F_{7, 192} = 12.5$ ($p = 0.003$)	$F_{7, 192} = 51.7$ ($p < 0.001$)
G × S × A × T	$F_{14, 192} = 14.2$ ($p < 0.001$)	$F_{14, 192} = 13.8$ ($p = 0.001$)	$F_{14, 192} = 6.6$ ($p = 0.004$)	$F_{14, 192} = 38.0$ ($p < 0.001$)

$p < 0.05$ was considered statistically significant.

Table S2. Factorial ANOVA of the four tested variables (maize genotype, aphid species, aphid abundance, infestation time), and the interactions on level of GSH/GSSG and AsA/DHA ratios in the seedling leaves of maize.

Tested Factors and Interactions	GSH/GSSG Ratio	AsA/DHA Ratio
Maize genotype (G)	$F_{1, 192} = 779.0$ ($p < 0.001$)	$F_{1, 192} = 1607.2$ ($p < 0.001$)
Aphid species (S)	$F_{2, 192} = 5601.5$ ($p < 0.001$)	$F_{2, 192} = 550.4$ ($p < 0.001$)
Aphid abundance (A)	$F_{1, 192} = 169.4$ ($p < 0.001$)	$F_{1, 192} = 886.2$ ($p < 0.001$)
Infestation time (T)	$F_{7, 192} = 593.2$ ($p < 0.001$)	$F_{7, 192} = 965.1$ ($p < 0.001$)
G × S	$F_{2, 192} = 144.0$ ($p < 0.001$)	$F_{2, 192} = 454.8$ ($p < 0.001$)
S × A	$F_{2, 192} = 175.3$ ($p < 0.001$)	$F_{2, 192} = 244.6$ ($p < 0.001$)
G × A	$F_{1, 192} = 265.8$ ($p < 0.001$)	$F_{1, 192} = 76.0$ ($p < 0.001$)
S × T	$F_{14, 192} = 91.2$ ($p < 0.001$)	$F_{14, 192} = 53.4$ ($p < 0.001$)
G × T	$F_{7, 192} = 302.7$ ($p < 0.001$)	$F_{7, 192} = 48.5$ ($p < 0.001$)
A × T	$F_{7, 192} = 194.6$ ($p < 0.001$)	$F_{7, 192} = 74.9$ ($p < 0.001$)
G × S × A	$F_{2, 192} = 66.9$ ($p < 0.001$)	$F_{2, 192} = 50.6$ ($p < 0.001$)
G × S × T	$F_{14, 192} = 110.3$ ($p < 0.001$)	$F_{14, 192} = 19.7$ ($p < 0.001$)
S × A × T	$F_{14, 192} = 82.2$ ($p < 0.001$)	$F_{14, 192} = 25.4$ ($p < 0.001$)
G × A × T	$F_{7, 192} = 31.5$ ($p < 0.001$)	$F_{7, 192} = 18.6$ ($p < 0.001$)
G × S × A × T	$F_{14, 192} = 20.4$ ($p < 0.001$)	$F_{14, 192} = 12.5$ ($p = 0.002$)

$p < 0.05$ was considered statistically significant.

Table S3. Factorial ANOVA of the four examined variables (maize genotype, aphid species, aphid abundance, infestation time), and their interactions on relative expression of *APX1*, *APX2*, *APX3* and *APX4* genes in the seedling leaves of maize (first phase of the experiments).

Tested Factors and Interactions	*APX1*	*APX2*	*APX3*	*APX4*
Maize genotype (G)	$F_{1, 192}$ = 2123.8 (p < 0.001)	$F_{1, 192}$ = 1025.6 (p < 0.001)	$F_{1, 192}$ = 1.3 (p = 0.305 ^{ns})	$F_{1, 192}$ = 1393.5 (p < 0.001)
Aphid species (S)	$F_{2, 192}$ = 1054.2 (p < 0.001)	$F_{2, 192}$ = 1403.1 (p < 0.001)	$F_{2, 192}$ = 2.2 (p = 0.412 ^{ns})	$F_{2, 192}$ = 723.6 (p < 0.001)
Aphid abundance (A)	$F_{1, 192}$ = 231.9 (p < 0.001)	$F_{1, 192}$ = 752.8 (p < 0.001)	$F_{1, 192}$ = 3.1 (p = 0.062 ^{ns})	$F_{1, 192}$ = 419.5 (p < 0.001)
Infestation time (T)	$F_{7, 192}$ = 615.4 (p < 0.001)	$F_{7, 192}$ = 503.1 (p < 0.001)	$F_{7, 192}$ = 1.5 (p = 0.348 ^{ns})	$F_{7, 192}$ = 573.2 (p < 0.001)
G × S	$F_{2, 192}$ = 550.8 (p < 0.001)	$F_{2, 192}$ = 353.8 (p < 0.001)	$F_{2, 192}$ = 6.2 (p = 0.075 ^{ns})	$F_{2, 192}$ = 349.2 (p < 0.001)
S × A	$F_{2, 192}$ = 60.7 (p < 0.001)	$F_{2, 192}$ = 190.5 (p < 0.001)	$F_{2, 192}$ = 1.0 (p = 0.283 ^{ns})	$F_{2, 192}$ = 104.9 (p < 0.001)
G × A	$F_{1, 192}$ = 82.3 (p < 0.001)	$F_{1, 192}$ = 353.6 (p < 0.001)	$F_{1, 192}$ = 1.2 (p = 0.269 ^{ns})	$F_{1, 192}$ = 196.0 (p < 0.001)
S × T	$F_{14, 192}$ = 159.6 (p < 0.001)	$F_{14, 192}$ = 135.8 (p < 0.001)	$F_{14, 192}$ = 4.8 (p = 0.120 ^{ns})	$F_{14, 192}$ = 145.7 (p < 0.001)
G × T	$F_{7, 192}$ = 274.1 (p < 0.001)	$F_{7, 192}$ = 151.8 (p < 0.001)	$F_{7, 192}$ = 5.7 (p = 0.054 ^{ns})	$F_{7, 192}$ = 242.3 (p < 0.001)
A × T	$F_{7, 192}$ = 36.3 (p < 0.001)	$F_{7, 192}$ = 113.6 (p < 0.001)	$F_{7, 192}$ = 2.4 (p = 0.076 ^{ns})	$F_{7, 192}$ = 95.2 (p < 0.001)
G × S × A	$F_{2, 192}$ = 20.6 (p < 0.001)	$F_{2, 192}$ = 89.1 (p < 0.001)	$F_{2, 192}$ = 0.5 (p = 0.610 ^{ns})	$F_{2, 192}$ = 49.1 (p < 0.001)
G × S × T	$F_{14, 192}$ = 73.9 (p < 0.001)	$F_{14, 192}$ = 40.3 (p < 0.001)	$F_{14, 192}$ = 1.8 (p = 0.064 ^{ns})	$F_{14, 192}$ = 62.9 (p < 0.001)
S × A × T	$F_{14, 192}$ = 11.8 (p < 0.001)	$F_{14, 192}$ = 29.0 (p < 0.001)	$F_{14, 192}$ = 0.6 (p = 0.832 ^{ns})	$F_{14, 192}$ = 26.4 (p < 0.001)
G × A × T	$F_{7, 192}$ = 23.6 (p < 0.001)	$F_{7, 192}$ = 52.6 (p < 0.001)	$F_{7, 192}$ = 0.3 (p = 0.955 ^{ns})	$F_{7, 192}$ = 43.4 (p < 0.001)
G × S × A × T	$F_{14, 192}$ = 9.4 (p < 0.001)	$F_{14, 192}$ = 17.7 (p < 0.001)	$F_{14, 192}$ = 0.2 (p = 0.991 ^{ns})	$F_{14, 192}$ = 12.8 (p < 0.001)

$p < 0.05$ was considered statistically significant; ^{ns}—Values not significant.

Table S4. Factorial ANOVA of the four examined variables (maize genotype, aphid species, aphid abundance, infestation time), and their interactions on relative expression of *APX5*, *APX6* and *APX7* genes in the seedling leaves of maize (first phase of the experiments).

Tested Factors and Interactions	*APX5*	*APX6*	*APX7*
Maize genotype (G)	$F_{1, 192} = 1110.4$ ($p < 0.001$)	$F_{1, 192} = 314.5$ ($p < 0.001$)	$F_{1, 192} = 2140.2$ ($p < 0.001$)
Aphid species (S)	$F_{2, 192} = 599.7$ ($p < 0.001$)	$F_{2, 192} = 229.5$ ($p < 0.001$)	$F_{2, 192} = 1535.6$ ($p < 0.001$)
Aphid abundance (A)	$F_{1, 192} = 89.5$ ($p < 0.001$)	$F_{1, 192} = 64.3$ ($p < 0.001$)	$F_{1, 192} = 424.1$ ($p < 0.001$)
Infestation time (T)	$F_{7, 192} = 648.9$ ($p < 0.001$)	$F_{7, 192} = 171.5$ ($p < 0.001$)	$F_{7, 192} = 720.9$ ($p < 0.001$)
G × S	$F_{2, 192} = 290.3$ ($p < 0.001$)	$F_{2, 192} = 78.9$ ($p < 0.001$)	$F_{2, 192} = 535.1$ ($p < 0.001$)
S × A	$F_{2, 192} = 23.2$ ($p < 0.001$)	$F_{2, 192} = 22.6$ ($p = 0.010$)	$F_{2, 192} = 106.9$ ($p < 0.001$)
G × A	$F_{1, 192} = 3.9$ ($p = 0.024$)	$F_{1, 192} = 10.9$ ($p = 0.017$)	$F_{1, 192} = 33.6$ ($p < 0.001$)
S × T	$F_{14, 192} = 167.4$ ($p < 0.001$)	$F_{14, 192} = 44.6$ ($p < 0.001$)	$F_{14, 192} = 191.5$ ($p < 0.001$)
G × T	$F_{7, 192} = 470.4$ ($p < 0.001$)	$F_{7, 192} = 80.1$ ($p < 0.001$)	$F_{7, 192} = 409.9$ ($p < 0.001$)
A × T	$F_{7, 192} = 24.2$ ($p < 0.001$)	$F_{7, 192} = 8.4$ ($p = 0.008$)	$F_{7, 192} = 39.6$ ($p < 0.001$)
G × S × A	$F_{2, 192} = 1.6$ ($p = 0.258$ [ns])	$F_{2, 192} = 5.2$ ($p = 0.026$)	$F_{2, 192} = 18.2$ ($p < 0.001$)
G × S × T	$F_{14, 192} = 122.9$ ($p < 0.001$)	$F_{14, 192} = 20.5$ ($p < 0.001$)	$F_{14, 192} = 110.1$ ($p < 0.001$)
S × A × T	$F_{14, 192} = 10.3$ ($p = 0.027$)	$F_{14, 192} = 5.6$ ($p = 0.029$)	$F_{14, 192} = 14.5$ ($p < 0.001$)
G × A × T	$F_{7, 192} = 7.7$ ($p = 0.035$)	$F_{7, 192} = 4.3$ ($p = 0.034$)	$F_{7, 192} = 32.4$ ($p < 0.001$)
G × S × A × T	$F_{14, 192} = 4.9$ ($p = 0.041$)	$F_{14, 192} = 6.1$ ($p = 0.025$)	$F_{14, 192} = 14.8$ ($p < 0.001$)

$p < 0.05$ was considered statistically significant; [ns]—Values not significant.

Table S5. Factorial ANOVA of the three examined variables (maize genotype, aphid species, infestation time), and their interactions on relative expression of *APX1*, *MDHAR1*, *DHAR2* and *GR1* genes in the seedling leaves of maize (second phase of the experiments).

Tested Factors and Interactions	*APX1*	*MDHAR1*	*DHAR2*	*GR1*
Maize genotype (G)	$F_{5, 108}$ = 225.4 (p < 0.001)	$F_{5, 108}$ = 253.9 (p < 0.001)	$F_{5, 108}$ = 415.1 (p < 0.001)	$F_{5, 108}$ = 883.1 (p < 0.001)
Aphid species (S)	$F_{2, 108}$ = 510.6 (p < 0.001)	$F_{2, 108}$ = 1134.8 (p < 0.001)	$F_{2, 108}$ = 117.6 (p < 0.001)	$F_{2, 108}$ = 3155.0 (p < 0.001)
Infestation time (T)	$F_{2, 108}$ = 423.5 (p < 0.001)	$F_{2, 108}$ = 2935.0 (p < 0.001)	$F_{2, 108}$ = 1472.4 (p < 0.001)	$F_{2, 108}$ = 934.7 (p < 0.001)
G × S	$F_{10, 108}$ = 60.9 (p < 0.001)	$F_{10, 108}$ = 104.6 (p < 0.001)	$F_{10, 108}$ = 28.1 (p < 0.001)	$F_{10, 108}$ = 240.8 (p < 0.001)
S × T	$F_{4, 108}$ = 123.2 (p < 0.001)	$F_{4, 108}$ = 962.7 (p < 0.001)	$F_{4, 108}$ = 415.6 (p < 0.001)	$F_{4, 108}$ = 195.3 (p < 0.001)
G × T	$F_{10, 108}$ = 68.0 (p < 0.001)	$F_{10, 108}$ = 210.5 (p < 0.001)	$F_{10, 108}$ = 117.4 (p < 0.001)	$F_{10, 108}$ = 67.5 (p < 0.001)
G × S × T	$F_{20, 108}$ = 20.4 (p < 0.001)	$F_{20, 108}$ = 79.6 (p < 0.001)	$F_{20, 108}$ = 38.0 (p < 0.001)	$F_{20, 108}$ = 30.2 (p < 0.001)

$p < 0.05$ was considered statistically significant.

Table S6. Factorial ANOVA of the four examined variables (maize genotype, aphid species, aphid abundance, infestation time), and their interactions on total activity of APX, MDHAR, DHAR and GR enzymes in the seedling leaves of maize.

Tested Factors and Interactions	APX	MDHAR	DHAR	GR
Maize genotype (G)	$F_{1, 192} = 948.6$ ($p = 0.005$)	$F_{1, 192} = 552.5$ ($p < 0.001$)	$F_{1, 192} = 784.0$ ($p < 0.001$)	$F_{1, 192} = 4326.2$ ($p < 0.001$)
Aphid species (S)	$F_{2, 192} = 4090.3$ ($p < 0.001$)	$F_{2, 192} = 236.1$ ($p < 0.001$)	$F_{2, 192} = 165.4$ ($p < 0.001$)	$F_{2, 192} = 1512.4$ ($p < 0.001$)
Aphid abundance (A)	$F_{1, 192} = 177.5$ ($p < 0.001$)	$F_{1, 192} = 18.0$ ($p < 0.001$)	$F_{1, 192} = 39.7$ ($p < 0.001$)	$F_{1, 192} = 240.8$ ($p < 0.001$)
Infestation time (T)	$F_{7, 192} = 2150.2$ ($p < 0.001$)	$F_{7, 192} = 162.6$ ($p < 0.001$)	$F_{7, 192} = 225.4$ ($p < 0.001$)	$F_{7, 192} = 1230.5$ ($p < 0.001$)
G × S	$F_{2, 192} = 964.0$ ($p < 0.001$)	$F_{2, 192} = 17.1$ ($p = 0.004$)	$F_{2, 192} = 23.8$ ($p < 0.001$)	$F_{2, 192} = 465.6$ ($p < 0.001$)
S × A	$F_{2, 192} = 593.7$ ($p < 0.001$)	$F_{2, 192} = 1.2$ ($p = 0.327$ ^{ns})	$F_{2, 192} = 8.5$ ($p = 0.095$ ^{ns})	$F_{2, 192} = 18.1$ ($p = 0.030$)
G × A	$F_{1, 192} = 146.2$ ($p < 0.001$)	$F_{1, 192} = 0.9$ ($p = 0.724$ ^{ns})	$F_{1, 192} = 14.7$ ($p < 0.001$)	$F_{1, 192} = 0.6$ ($p = 0.874$ ^{ns})
S × T	$F_{14, 192} = 561.4$ ($p < 0.001$)	$F_{14, 192} = 19.5$ ($p < 0.001$)	$F_{14, 192} = 10.2$ ($p = 0.004$)	$F_{14, 192} = 311.4$ ($p < 0.001$)
G × T	$F_{7, 192} = 508.0$ ($p < 0.001$)	$F_{7, 192} = 23.8$ ($p < 0.001$)	$F_{7, 192} = 29.0$ ($p < 0.001$)	$F_{7, 192} = 282.7$ ($p < 0.001$)
A × T	$F_{7, 192} = 97.5$ ($p < 0.001$)	$F_{7, 192} = 13.5$ ($p = 0.001$)	$F_{7, 192} = 17.6$ ($p = 0.002$)	$F_{7, 192} = 15.3$ ($p = 0.002$)
G × S × A	$F_{2, 192} = 64.8$ ($p < 0.001$)	$F_{2, 192} = 0.8$ ($p = 0.418$ ^{ns})	$F_{2, 192} = 1.8$ ($p = 0.541$ ^{ns})	$F_{2, 192} = 0.2$ ($p = 0.910$ ^{ns})
G × S × T	$F_{14, 192} = 137.2$ ($p < 0.001$)	$F_{14, 192} = 1.2$ ($p = 0.270$ ^{ns})	$F_{14, 192} = 32.4$ ($p < 0.001$)	$F_{14, 192} = 80.7$ ($p < 0.001$)
S × A × T	$F_{14, 192} = 12.6$ ($p = 0.008$)	$F_{14, 192} = 2.6$ ($p = 0.194$ ^{ns})	$F_{14, 192} = 19.5$ ($p = 0.002$)	$F_{14, 192} = 1.6$ ($p = 0.075$ ^{ns})
G × A × T	$F_{7, 192} = 3.5$ ($p = 0.015$)	$F_{7, 192} = 1.5$ ($p = 0.386$ ^{ns})	$F_{7, 192} = 17.8$ ($p = 0.006$)	$F_{7, 192} = 0.5$ ($p = 0.908$ ^{ns})
G × S × A × T	$F_{14, 192} = 8.4$ ($p = 0.010$)	$F_{14, 192} = 0.6$ ($p = 0.512$ ^{ns})	$F_{14, 192} = 1.2$ ($p = 0.730$ ^{ns})	$F_{14, 192} = 0.3$ ($p = 0.946$ ^{ns})

$p < 0.05$ was considered statistically significant; ^{ns}—Values not significant.

Table S7. Factorial ANOVA of the four examined variables (maize genotype, aphid species, aphid abundance, infestation time), and their interactions on relative expression of *MDHAR1*, *MDHAR2*, *MDHAR3* and *MDHAR4* genes in the seedling leaves of maize (first phase of the experiments).

Tested Factors and Interactions	*MDHAR1*	*MDHAR2*	*MDHAR3*	*MDHAR4*
Maize genotype (G)	$F_{1, 192} = 969.2$ ($p < 0.001$)	$F_{1, 192} = 158.6$ ($p < 0.001$)	$F_{1, 192} = 38.6$ ($p < 0.001$)	$F_{1, 192} = 6.9$ ($p = 0.112$ ns)
Aphid species (S)	$F_{2, 192} = 2080.1$ ($p < 0.001$)	$F_{2, 192} = 114.1$ ($p < 0.001$)	$F_{2, 192} = 50.8$ ($p < 0.001$)	$F_{2, 192} = 9.7$ ($p = 0.296$ ns)
Aphid abundance (A)	$F_{1, 192} = 195.0$ ($p < 0.001$)	$F_{1, 192} = 58.9$ ($p < 0.001$)	$F_{1, 192} = 29.2$ ($p < 0.001$)	$F_{1, 192} = 8.1$ ($p = 0.124$ ns)
Infestation time (T)	$F_{7, 192} = 1165.8$ ($p < 0.001$)	$F_{7, 192} = 103.4$ ($p < 0.001$)	$F_{7, 192} = 77.9$ ($p < 0.001$)	$F_{7, 192} = 5.7$ ($p = 0.085$ ns)
G × S	$F_{2, 192} = 535.4$ ($p < 0.001$)	$F_{2, 192} = 42.5$ ($p < 0.001$)	$F_{2, 192} = 11.6$ ($p = 0.057$ ns)	$F_{2, 192} = 4.3$ ($p = 0.099$ ns)
S × A	$F_{2, 192} = 48.9$ ($p < 0.001$)	$F_{2, 192} = 15.8$ ($p = 0.024$)	$F_{2, 192} = 7.3$ ($p = 0.086$ ns)	$F_{2, 192} = 3.4$ ($p = 0.207$ ns)
G × A	$F_{1, 192} = 80.6$ ($p < 0.001$)	$F_{1, 192} = 12.9$ ($p = 0.016$)	$F_{1, 192} = 16.2$ ($p = 0.009$)	$F_{1, 192} = 3.9$ ($p = 0.164$ ns)
S × T	$F_{14, 192} = 298.0$ ($p < 0.001$)	$F_{14, 192} = 27.6$ ($p < 0.001$)	$F_{14, 192} = 20.4$ ($p = 0.015$)	$F_{14, 192} = 4.2$ ($p = 0.198$ ns)
G × T	$F_{7, 192} = 589.3$ ($p < 0.001$)	$F_{7, 192} = 112.4$ ($p < 0.001$)	$F_{7, 192} = 7.1$ ($p = 0.082$ ns)	$F_{7, 192} = 5.5$ ($p = 0.090$ ns)
A × T	$F_{7, 192} = 42.6$ ($p < 0.001$)	$F_{7, 192} = 14.5$ ($p = 0.022$)	$F_{7, 192} = 12.9$ ($p = 0.064$ ns)	$F_{7, 192} = 2.8$ ($p = 0.165$ ns)
G × S × A	$F_{2, 192} = 20.2$ ($p = 0.010$)	$F_{2, 192} = 16.6$ ($p = 0.034$)	$F_{2, 192} = 4.7$ ($p = 0.091$ ns)	$F_{2, 192} = 1.0$ ($p = 0.352$ ns)
G × S × T	$F_{14, 192} = 149.5$ ($p < 0.001$)	$F_{14, 192} = 29.0$ ($p < 0.001$)	$F_{14, 192} = 5.0$ ($p = 0.105$ ns)	$F_{14, 192} = 1.4$ ($p = 0.468$ ns)
S × A × T	$F_{14, 192} = 12.1$ ($p = 0.028$)	$F_{14, 192} = 4.5$ ($p = 0.098$ ns)	$F_{14, 192} = 3.5$ ($p = 0.167$ ns)	$F_{14, 192} = 0.8$ ($p = 0.696$ ns)
G × A × T	$F_{7, 192} = 33.9$ ($p < 0.001$)	$F_{7, 192} = 17.1$ ($p < 0.001$)	$F_{7, 192} = 9.3$ ($p = 0.085$ ns)	$F_{7, 192} = 1.9$ ($p = 0.084$ ns)
G × S × A × T	$F_{14, 192} = 10.6$ ($p = 0.035$)	$F_{14, 192} = 4.9$ ($p < 0.001$ ns)	$F_{14, 192} = 2.4$ ($p = 0.346$ ns)	$F_{14, 192} = 0.5$ ($p = 0.932$ ns)

$p < 0.05$ was considered statistically significant; ns—Values not significant.

Table S8. Factorial ANOVA of the four examined variables (maize genotype, aphid species, aphid abundance, infestation time), and their interactions on relative expression of *DHAR1*, *DHAR2* and *DHAR3* genes in the seedling leaves of maize (first phase of the experiments).

Tested Factors and Interactions	*DHAR1*	*DHAR2*	*DHAR3*
Maize genotype (G)	$F_{1, 192}$ = 8.2 (p = 0.045)	$F_{1, 192}$ = 334.7 (p < 0.001)	$F_{1, 192}$ = 1.1 (p = 0.516 ^{ns})
Aphid species (S)	$F_{2, 192}$ = 5.1 (p = 0.093 ^{ns})	$F_{2, 192}$ = 296.1 (p < 0.001)	$F_{2, 192}$ = 1.5 (p = 0.368 ^{ns})
Aphid abundance (A)	$F_{1, 192}$ = 12.6 (p = 0.009)	$F_{1, 192}$ = 101.2 (p < 0.001)	$F_{1, 192}$ = 2.7 (p = 0.220 ^{ns})
Infestation time (T)	$F_{7, 192}$ = 4.3 (p = 0.148 ^{ns})	$F_{7, 192}$ = 568.0 (p < 0.001)	$F_{7, 192}$ = 0.8 (p = 0.660 ^{ns})
G × S	$F_{2, 192}$ = 2.0 (p = 0.352 ^{ns})	$F_{2, 192}$ = 85.3 (p < 0.001)	$F_{2, 192}$ = 1.2 (p = 0.541 ^{ns})
S × A	$F_{2, 192}$ = 1.5 (p = 0.463 ^{ns})	$F_{2, 192}$ = 25.8 (p = 0.024)	$F_{2, 192}$ = 0.6 (p = 0.815 ^{ns})
G × A	$F_{1, 192}$ = 1.9 (p = 0.570 ^{ns})	$F_{1, 192}$ = 46.5 (p = 0.016)	$F_{1, 192}$ = 1.4 (p = 0.598 ^{ns})
S × T	$F_{14, 192}$ = 2.4 (p = 0.145 ^{ns})	$F_{14, 192}$ = 147.3 (p < 0.001)	$F_{14, 192}$ = 0.5 (p = 0.746 ^{ns})
G × T	$F_{7, 192}$ = 1.6 (p = 0.510 ^{ns})	$F_{7, 192}$ = 112.4 (p < 0.001)	$F_{7, 192}$ = 1.2 (p = 0.525 ^{ns})
A × T	$F_{7, 192}$ = 3.8 (p = 0.231 ^{ns})	$F_{7, 192}$ = 29.6 (p < 0.001)	$F_{7, 192}$ = 1.9 (p = 0.694 ^{ns})
G × S × A	$F_{2, 192}$ = 1.5 (p = 0.746 ^{ns})	$F_{2, 192}$ = 9.5 (p = 0.004)	$F_{2, 192}$ = 1.0 (p = 0.632 ^{ns})
G × S × T	$F_{14, 192}$ = 2.6 (p = 0.218 ^{ns})	$F_{14, 192}$ = 35.4 (p < 0.001)	$F_{14, 192}$ = 0.3 (p = 0.851 ^{ns})
S × A × T	$F_{14, 192}$ = 1.2 (p = 0.565 ^{ns})	$F_{14, 192}$ = 8.1 (p = 0.001)	$F_{14, 192}$ = 0.9 (p = 0.742 ^{ns})
G × A × T	$F_{7, 192}$ = 0.8 (p = 0.422 ^{ns})	$F_{7, 192}$ = 7.0 (p = 0.002)	$F_{7, 192}$ = 0.4 (p = 0.790 ^{ns})
G × S × A × T	$F_{14, 192}$ = 0.6 (p = 0.350 ^{ns})	$F_{14, 192}$ = 3.4 (p = 0.006)	$F_{14, 192}$ = 0.2 (p = 0.915 ^{ns})

p < 0.05 was considered statistically significant; ^{ns}—Values not significant.

Table S9. Factorial ANOVA of the four examined variables (maize genotype, aphid species, aphid abundance, infestation time), and their interactions on relative expression of *GR1* and *GR2* genes in the seedling leaves of maize (first phase of the experiments).

Tested Factors and Interactions	*GR1*	*GR2*
Maize genotype (G)	$F_{1,192} = 1143.2$ ($p < 0.001$)	$F_{1,192} = 124.8$ ($p < 0.001$)
Aphid species (S)	$F_{2,192} = 545.8$ ($p < 0.001$)	$F_{2,192} = 84.9$ ($p < 0.001$)
Aphid abundance (A)	$F_{1,192} = 107.0$ ($p < 0.001$)	$F_{1,192} = 26.6$ ($p < 0.001$)
Infestation time (T)	$F_{7,192} = 361.5$ ($p < 0.001$)	$F_{7,192} = 71.2$ ($p < 0.001$)
G × S	$F_{2,192} = 298.3$ ($p < 0.001$)	$F_{2,192} = 40.3$ ($p < 0.001$)
S × A	$F_{2,192} = 27.4$ ($p < 0.001$)	$F_{2,192} = 9.5$ ($p = 0.006$)
G × A	$F_{1,192} = 45.9$ ($p < 0.001$)	$F_{1,192} = 6.8$ ($p = 0.025$)
S × T	$F_{14,192} = 103.2$ ($p < 0.001$)	$F_{14,192} = 20.9$ ($p < 0.001$)
G × T	$F_{7,192} = 174.5$ ($p < 0.001$)	$F_{7,192} = 25.4$ ($p < 0.001$)
A × T	$F_{7,192} = 14.8$ ($p < 0.001$)	$F_{7,192} = 5.8$ ($p = 0.189$ ^{ns})
G × S × A	$F_{2,192} = 12.0$ ($p < 0.001$)	$F_{2,192} = 1.4$ ($p = 0.105$ ^{ns})
G × S × T	$F_{14,192} = 49.2$ ($p < 0.001$)	$F_{14,192} = 10.6$ ($p = 0.003$)
S × A × T	$F_{14,192} = 9.7$ ($p = 0.012$)	$F_{14,192} = 2.1$ ($p = 0.215$ ^{ns})
G × A × T	$F_{7,192} = 6.5$ ($p = 0.006$)	$F_{7,192} = 1.1$ ($p = 0.342$ ^{ns})
G × S × A × T	$F_{14,192} = 8.1$ ($p = 0.002$)	$F_{14,192} = 0.7$ ($p = 0.826$ ^{ns})

$p < 0.05$ was considered statistically significant; ^{ns}—Values not significant.

Table S10. List of the target maize genes and *TaqMan Gene Expression Assays* [a].

Target Gene (Gene Locus) [b]	*GenBank* Reference Sequence	Subcellular Localisation [c]	Assay ID
APX1 (GRMZM2G054300)	NM_001156720.1	Cytosolic	Zm04073938_gH
APX2 (GRMZM2G140667)	NM_001112030.1	Cytosolic	Zm04052467_g1
APX3 (GRMZM2G004211)	NM_001155238.1	Peroxisomal	Zm04044555_g1
APX4 (GRMZM2G460406)	NM_001139033.1	Cytosolic	Zm04070610_m1
APX5 (GRMZM2G014397)	NM_001138827.1	Mitochondrial	Zm04066499_m1
APX6 (GRMZM2G120517)	NM_001139211.1	Mitochondrial	Zm04067659_m1
APX7 (*t-APX8*) (GRMZM2G006791)	NM_001156037.1	Chloroplastic (thylakoids)	Zm04039568_g1

[a] *TaqMan Gene Expression Assays* were purchased from Life Technologies, Poland (www.lifetechnologies.com); [b] Gene locus was itemized according to the Maize Genetics and Genomics Database— MaizeGDB (http://www.maizegdb.org; accessed on 5 March 2013); [c] Subcellular localisation of the encoded isozymes was specified as described by Liu *et al.* [16].

Table S11. List of primers designed for the analysed maize genes [a].

Target Gene	Gene Locus [b]	Subcellular Localisation [c]	Sequence of Primer
MDHAR1	GRMZM2G084881	Cytosolic	F: TCAAGGGTCAAGTTGCTGAG<br>R: AGGGAAGGTAGCCACATCAC
MDHAR2	GRMZM2G134708	Cytosolic	F: CTCCGACTCTTCCATTCCAT<br>R: TTGAAGTGCCTAGCCATCAC
MDHAR3	GRMZM2G320307	Cytosolic	F: GGAAATGCTGTTGTCATTGG<br>R: TGCAGTGTTTCTCAGGGAAG
MDHAR4	GRMZM5G828229	Mitochondrial	F: TGGAGGGAAATTACCTGGAG<br>R: TGCACTTCCCAATGAGGATA
DHAR1	GRMZM2G005710	Chloroplastic	F: CGACTGTCCCTTCTCACAAA<br>R: ATCCGGTAAGGGACCTTCTT
DHAR2	GRMZM5G855672	Cytosolic	F: GAATTATGCAGTGCCGTGTC<br>R: GCTCTCACAGCATAGGACCA
DHAR3	GRMZM5G826194	Mitochondrial	F: TAACGCGCGAGTGTGTAAAT<br>R: TCCAAAGTACGACCCTACCC
GR1	GRMZM2G172322	Cytosolic	F: TTGGCAATGAACCTACCAAA<br>R: CAATTGCCTGCTCCTCAGTA
GR2	GRMZM5G806449	Cytosolic	F: TACGATCTCTTCGTCATCGG<br>R: GCTCACAAATCGCAACCTTA

[a] *Custom TaqMan Gene Expression Assays* were purchased from Life Technologies, Poland (www.lifetechnologies.com). Sequences of the primers were designed using the online GenScript software (http://www.genscript.com/ssl-bin/app/primer; accessed on 5 March 2013); [b] Gene locus was itemized according to the Maize Genetics and Genomics Database—MaizeGDB (http://www.maizegdb.org; accessed on 5 March 2013); [c] Subcellular localisation of the encoded isozymes was specified as described by Liu *et al.* 2012 [16]. F—forward primer; R—reverse primer.

Table S12. List of *TaqMan* fluorescence probes designed for the analysed maize genes [a].

Target Gene	Sequence of *TaqMan* Fluorescent Probe
MDHAR1	5′-FAM-CGCTTCCTTTGAAACAAGCGTTCC-BBQ-3′
MDHAR2	5′-FAM-TTTCTTCCCGCAGCCTGCCT-BBQ-3′
MDHAR3	5′-FAM-CAATGCCGCTGCACACTCCA-BBQ-3′
MDHAR4	5′-FAM-CATCAGCATCAGCAACATCCCG-BBQ-3′
DHAR1	5′-FAM-TCCTCCAGAGTGAGCAGCACCC-BBQ-3′
DHAR2	5′-FAM-TGCACAACCGCAACAGCCAG-BBQ-3′
DHAR3	5′-FAM-CATCCATGCAGCAGACGGCA-BBQ-3′
GR1	5′-FAM-TGCTGTGTTCTCCCAACCACCA-BBQ-3′
GR2	5′-FAM-CGTTCGCGGCTCTCGCAC-BBQ-3′

[a] *Custom TaqMan Gene Expression Assays* were purchased from Life Technologies, Poland (www.lifetechnologies.com). Sequences of the fluorescent probes were designed using the online GenScript software (https://www.genscript.com/ssl-bin/app/primer; accessed on 5 March 2013).

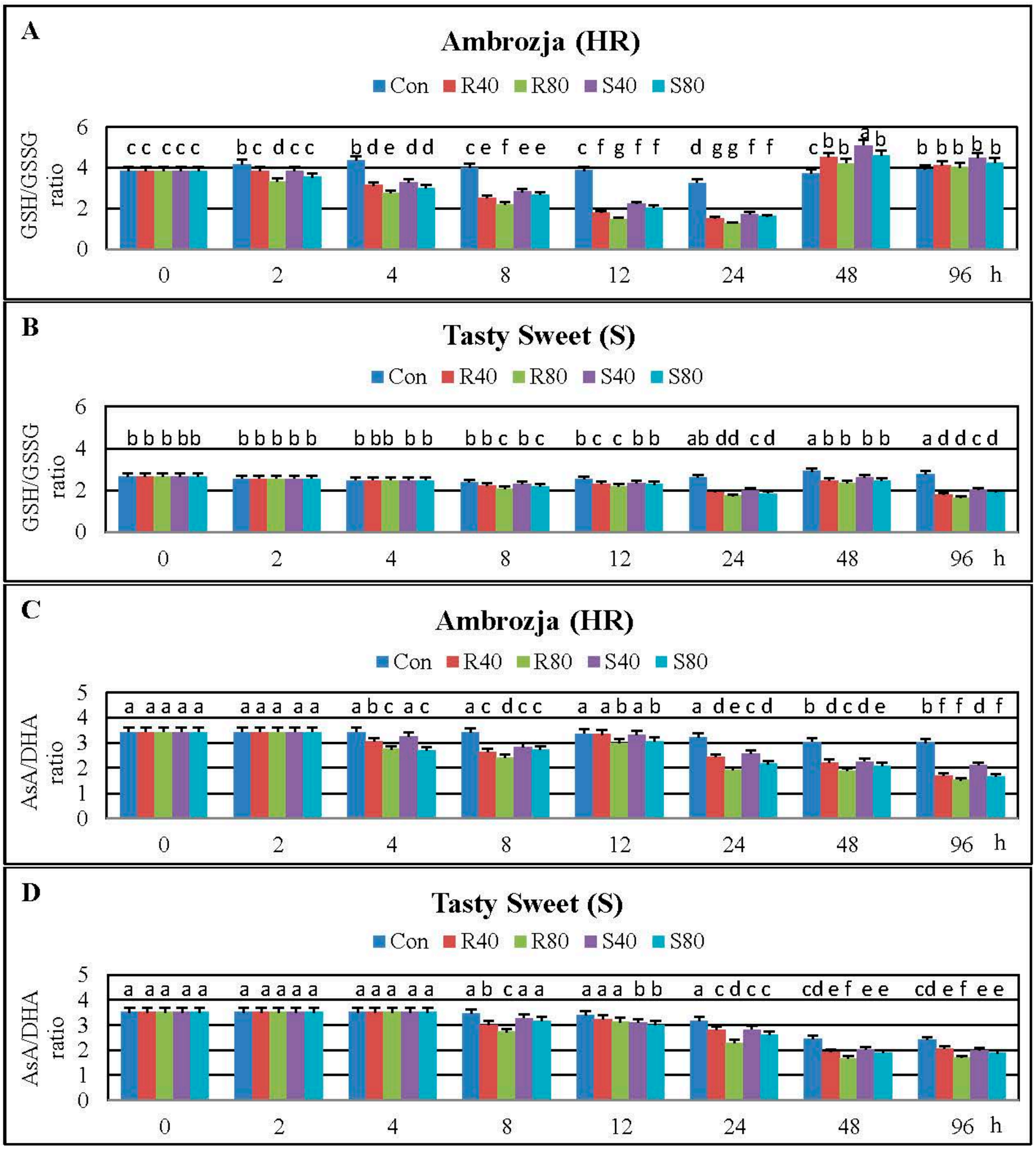

Figure S1. Impact of the cereal aphids' infestation on GSH/GSSG (**A**,**B**) and AsA/DHA (**C**,**D**) ratios in the seedlings of Ambrozja and Tasty Sweet genotypes. Hpi–hours post initial aphid infestation; HR—high resistance level to cereal aphids' attack; S—susceptible to the cereal aphids; Con —seedlings of a given maize cultivar uninfested with the hemipterans; R40, R80—maize plants of a certain genotype infested with 40 or 80 *R. padi* females per seedling, accordingly; S40, S80—maize plants of a certain genotype infested with 40 or 80 *S. avenae* females per seedling, respectively. All data are presented as the mean (±SD) of three independent biotests. Significant differences in level of GSH/GSSG and AsA/DHA ratios between tested groups of the maize plants are denoted by different letters ($p < 0.05$; *post-hoc* Tukey's test).

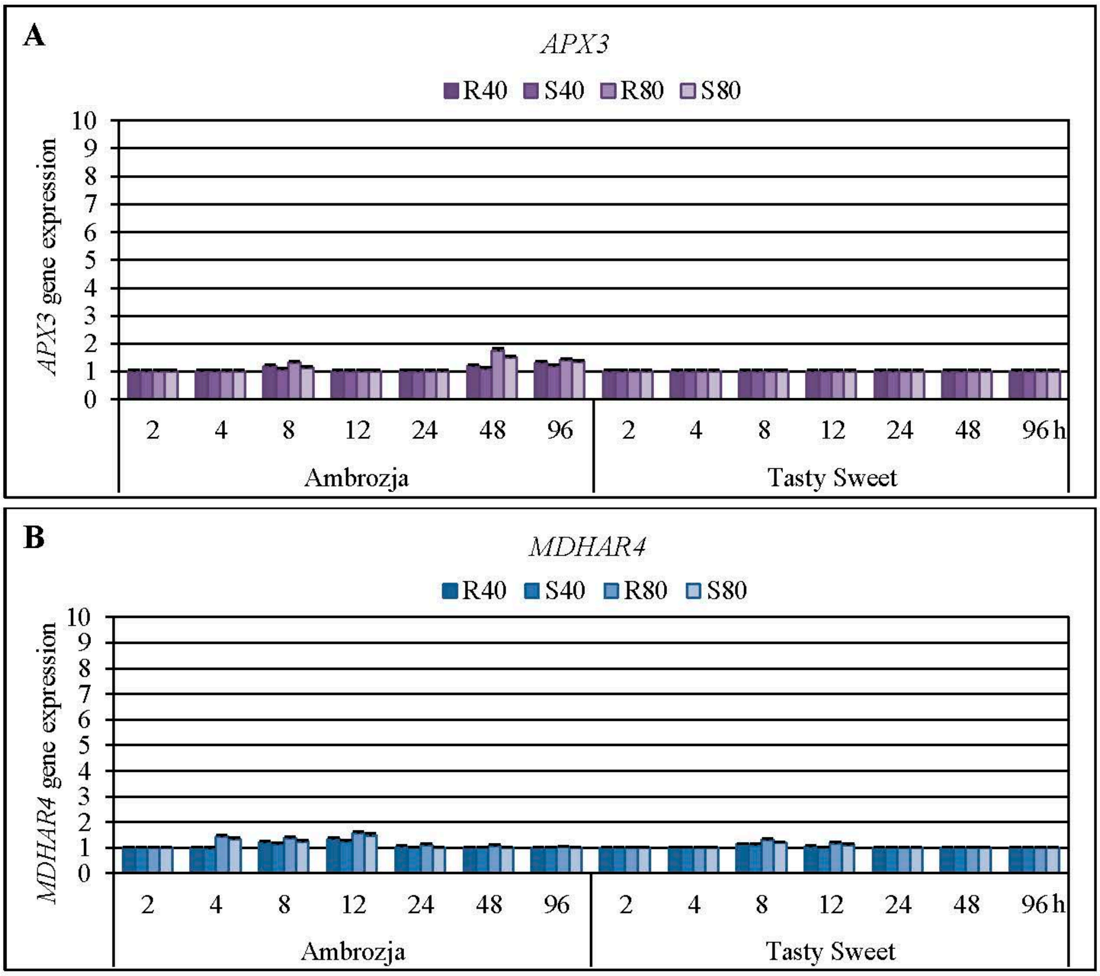

Figure S2. Influence of the cereal aphid's infestation on relative expression of *APX3* (**A**) and *MDHAR4* (**B**) genes in the seedlings of Ambrozja and Tasty Sweet maize varieties (first phase of the experiments). R40, R80—maize plants of a certain genotype infested with 40 or 80 *R. padi* females per seedling, accordingly; S40, S80—maize plants of a certain genotype infested with 40 or 80 *S. avenae* females per seedling, respectively. All data are presented as the mean (±SD) of three independent biotests. There were no differences in abundance of the studied transcripts between the tested groups of maize plants ($p < 0.05$; *post-hoc* Tukey's test).

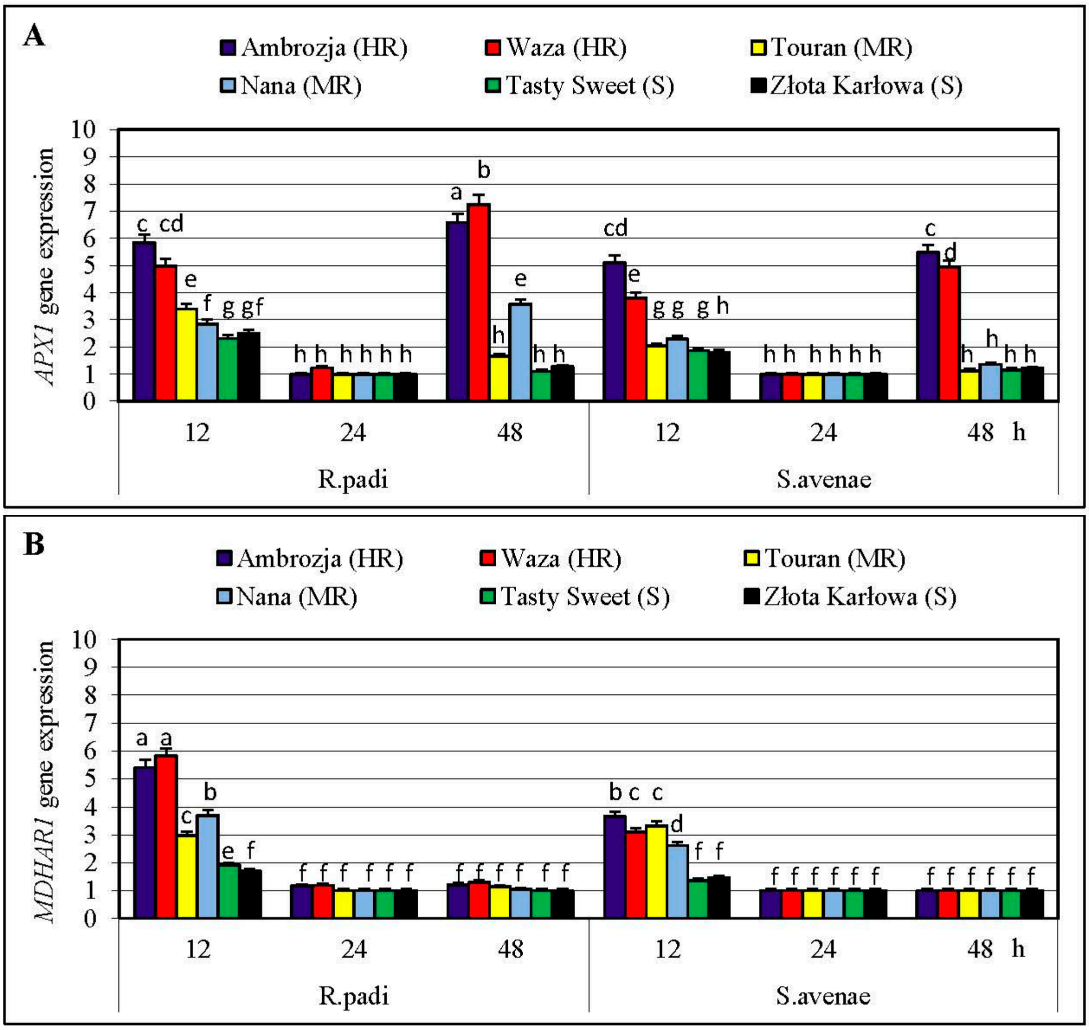

Figure S3. Effect of the cereal aphids' herbivory on relative expression of *APX1* (**A**) and *MDHAR1* (**B**) genes in *Z. mays* seedlings of Ambrozja, Waza, Touran, Nana, Tasty Sweet and Złota Karłowa varieties (second phase of the experiments). HR—high resistance level to cereal aphids; MR – intermediate resistance; S—susceptible to the cereal aphids. Maize plants of a certain genotype were infested with 100 *R. padi* or *S. avenae* females per seedling. All data are presented as the mean (±SD) of three independent biotests, and significant differences in abundance of the target transcripts are denoted by different letters ($p < 0.05$; *post-hoc* Tukey's test).

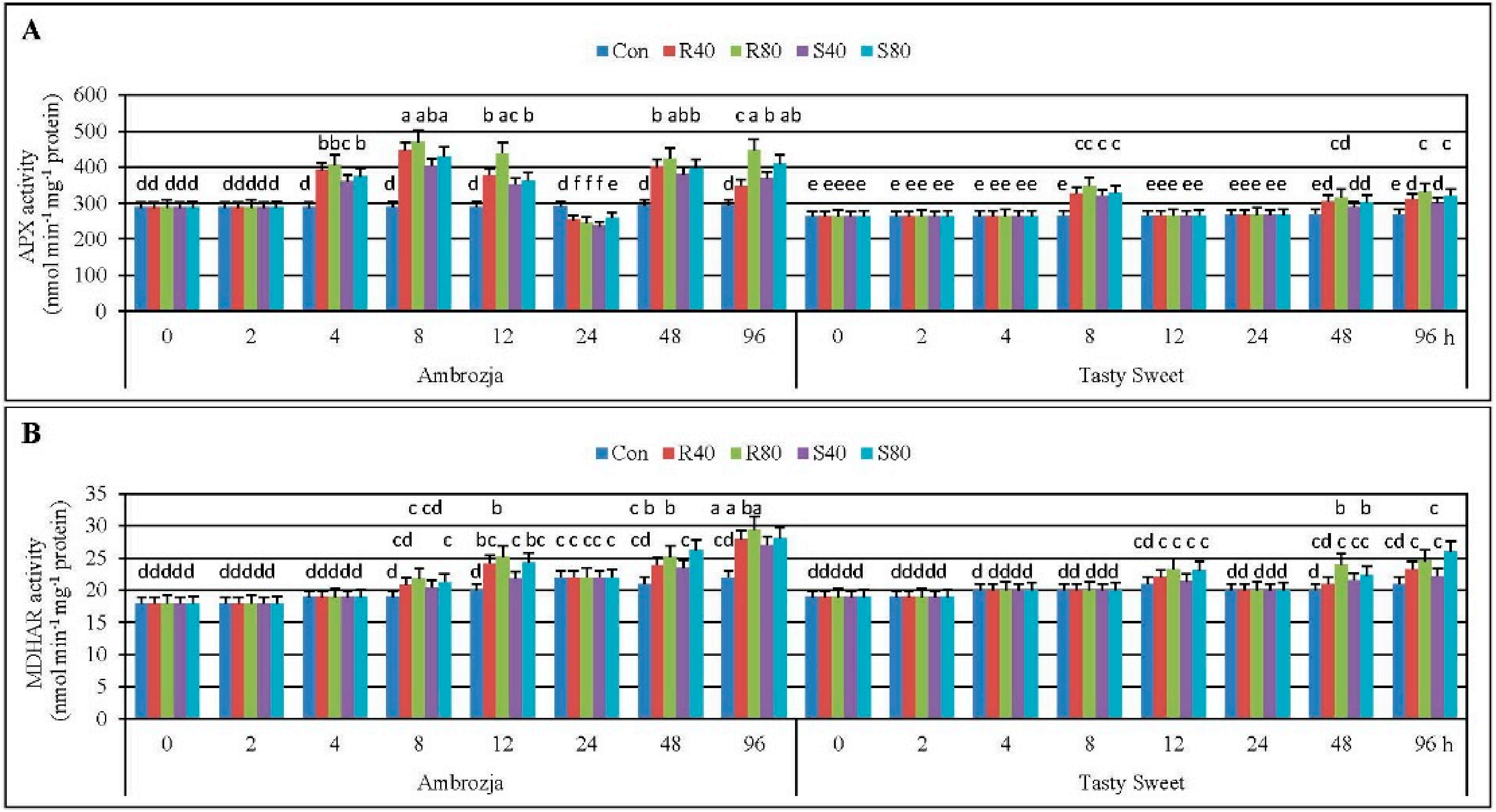

Figure S4. Impact of the cereal aphid's infestation on total activity of APX (**A**) and MDHAR (**B**) enzymes in the seedlings of Ambrozja and Tasty Sweet maize varieties. R40, R80—maize plants of a certain genotype infested with 40 or 80 *R. padi* females per seedling, accordingly; S40, S80—maize plants of a certain genotype infested with 40 or 80 *S. avenae* females per seedling, respectively. All data are presented as the mean (±SD) of three independent biotests, and significant differences in activity of the examined enzymes are denoted by different letters ($p < 0.05$; *post-hoc* Tukey's test).

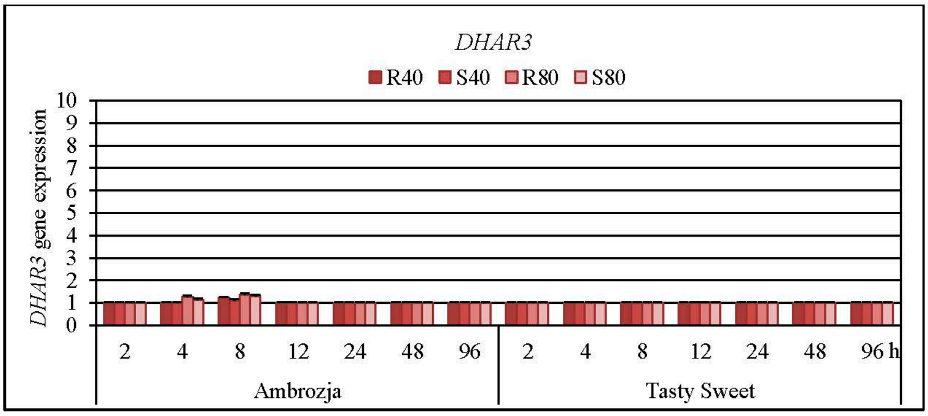

Figure S5. Impact of the cereal aphid's infestation on relative expression of *DHAR3* gene in the seedlings of Ambrozja and Tasty Sweet maize varieties (first phase of the experiments). R40, R80—maize plants of a certain genotype infested with 40 or 80 *R. padi* females per seedling, accordingly; S40, S80—maize plants of a certain genotype infested with 40 or 80 *S. avenae* females per seedling, respectively. All data are presented as the mean (±SD) of three independent biotests. There were no differences in abundance of the studied transcript between the tested groups of maize plants ($p < 0.05$; *post-hoc* Tukey's test).

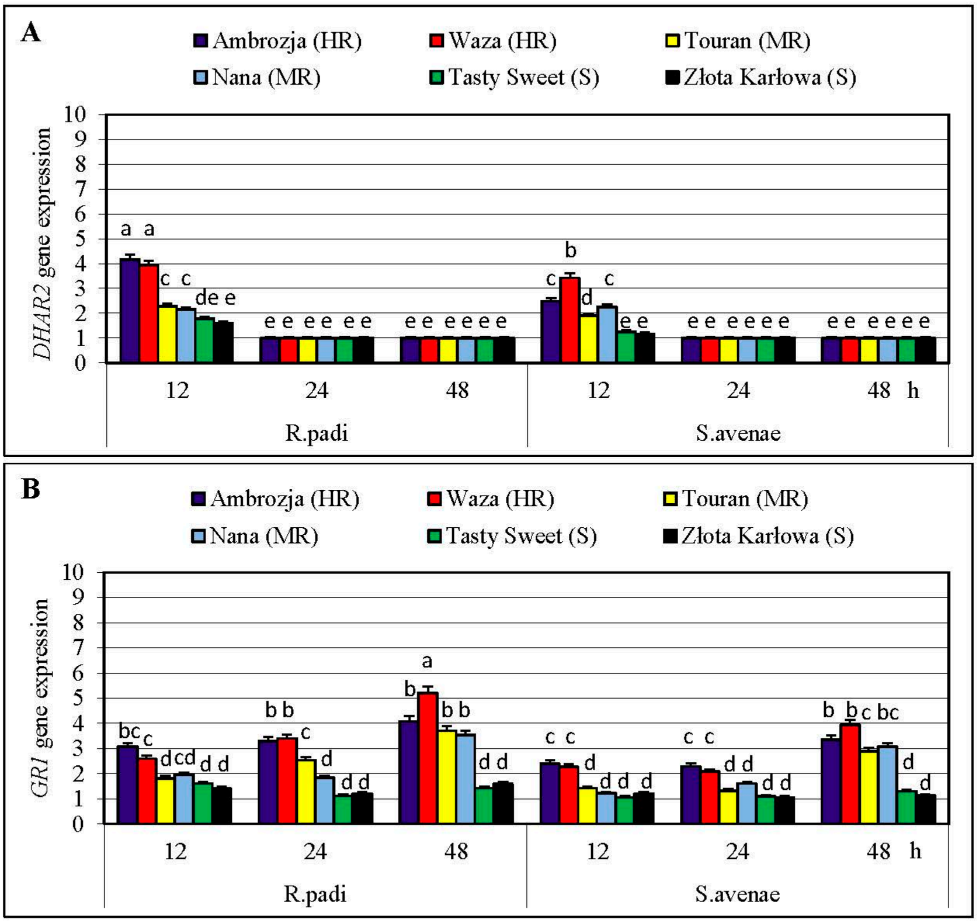

Figure S6. Effect of the cereal aphids' herbivory on relative expression of *DHAR2* (**A**) and *GR1* (**B**) genes in *Z. mays* seedlings of Ambrozja, Waza, Touran, Nana, Tasty Sweet and Złota Karłowa varieties (second phase of the experiments). HR—high resistance level to cereal aphids; MR—intermediate resistance; S—susceptible to the cereal aphids. Maize plants of a certain genotype were infested with 100 *R. padi* or *S. avenae* females per seedling. All data are presented as the mean (±SD) of three independent biotests, and significant differences in abundance of the target transcripts are denoted by different letters ($p < 0.05$; *post-hoc* Tukey's test).

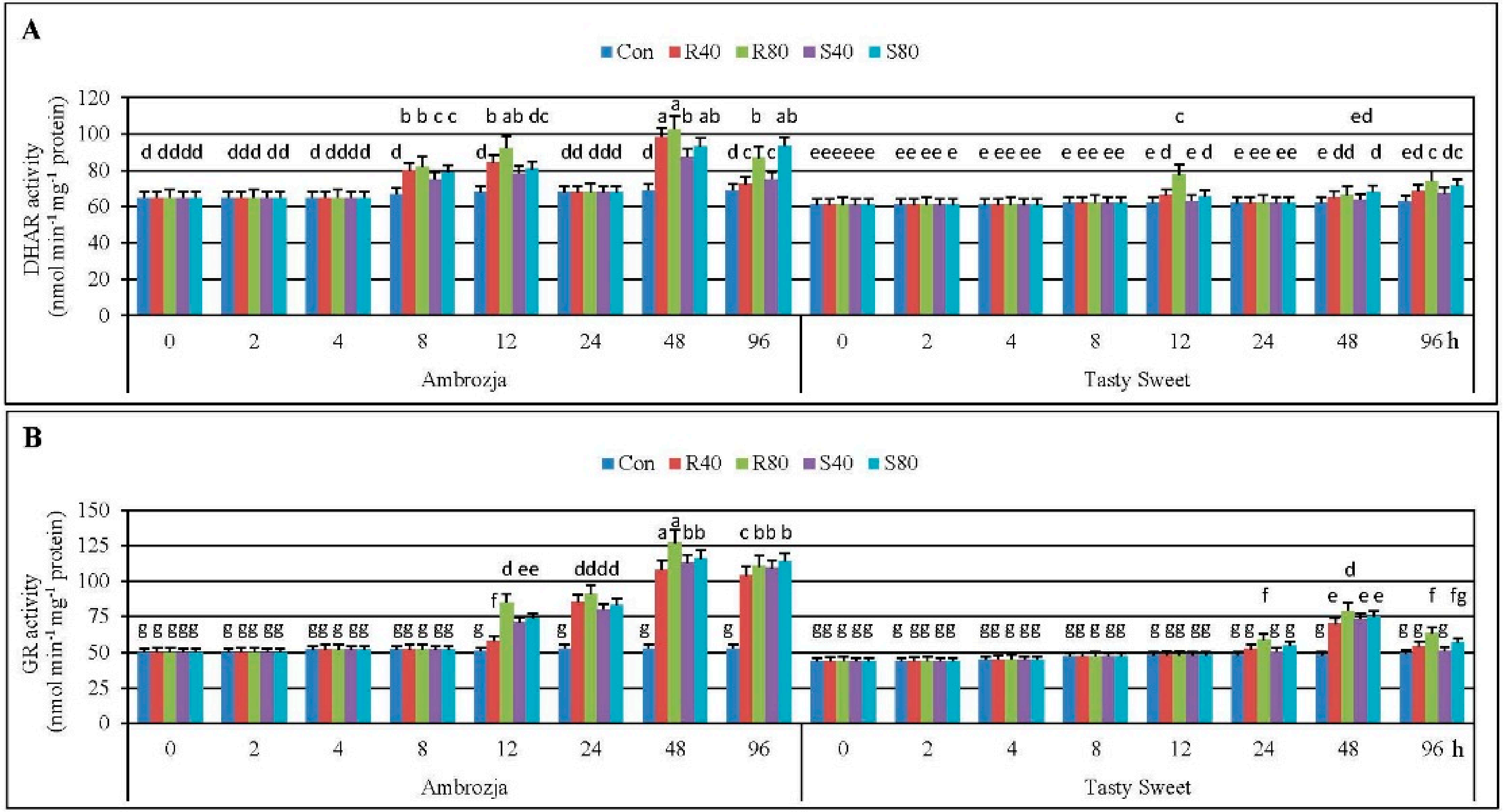

Figure S7. Impact of the cereal aphid's infestation on total activity of DHAR (**A**) and GR (**B**) enzymes in the seedlings of Ambrozja and Tasty Sweet maize varieties. R40, R80—maize plants of a certain genotype infested with 40 or 80 *R. padi* females per seedling, accordingly; S40, S80—maize plants of a certain genotype infested with 40 or 80 *S. avenae* females per seedling, respectively. All data are presented as the mean (±SD) of three independent biotests, and significant differences in activity of theexamined enzymes are denoted by different letters ($p < 0.05$; *post-hoc* Tukey's test)
